# Insight into Bortezomib Focusing on Its Efficacy against P-gp-Positive MDR Leukemia Cells

**DOI:** 10.3390/ijms22115504

**Published:** 2021-05-23

**Authors:** Tomáš Kyca, Lucia Pavlíková, Viera Boháčová, Anton Mišák, Alexandra Poturnayová, Albert Breier, Zdena Sulová, Mário Šereš

**Affiliations:** 1 Institute of Molecular Physiology and Genetics, Centre of Biosciences, Slovak Academy of Sciences, Dúbravská cesta 9, 84505 Bratislava, Slovakia; tomas.kyca@savba.sk (T.K.); lucia.pavlikova@savba.sk (L.P.); viera.bohacova@savba.sk (V.B.); alexandra.poturnayova@savba.sk (A.P.); 2 Institute of Zoology, Slovak Academy of Sciences, Dúbravská cesta 9, 84506 Bratislava, Slovakia; 3 Institute for Clinical and Translational Research, Biomedical Research Center, Slovak Academy of Sciences, Dúbravská cesta 9, 84505 Bratislava, Slovakia; anton.misak@savba.sk; 4 Institute of Biochemistry and Microbiology, Faculty of Chemical and Food Technology, Slovak University of Technology in Bratislava, Radlinského 9, 81237 Bratislava 1, Slovakia

**Keywords:** bortezomib, P-glycoprotein, L1210 cells, cyclin-dependent kinases, cyclins, CDK inhibitors, ubiquitination, deubiquitinases, 26S proteasome, HSP90

## Abstract

In this paper, we compared the effects of bortezomib on L1210 (S) cells with its effects on P-glycoprotein (P-gp)-positive variant S cells, which expressed P-gp either after selection with vincristine (R cells) or after transfection with a human gene encoding P-gp (T cells). Bortezomib induced the death-related effects in the S, R, and T cells at concentrations not exceeding 10 nM. Bortezomib-induced cell cycle arrest in the G2/M phase was more pronounced in the S cells than in the R or T cells and was related to the expression levels of cyclins, cyclin-dependent kinases, and their inhibitors. We also observed an increase in the level of polyubiquitinated proteins (via K48-linkage) and a decrease in the gene expression of some deubiquitinases after treatment with bortezomib. Resistant cells expressed higher levels of genes encoding 26S proteasome components and the chaperone HSP90, which is involved in 26S proteasome assembly. After 4 h of preincubation, bortezomib induced a more pronounced depression of proteasome activity in S cells than in R or T cells. However, none of these changes alone or in combination sufficiently suppressed the sensitivity of R or T cells to bortezomib, which remained at a level similar to that of S cells.

## 1. Introduction

Bortezomib (BOR, for the chemical structure, see Figure S1 in the supplementary files) is an approved drug (FDA and EMA [1]) for the treatment of progressive multiple myeloma and mantle cell lymphoma in patients for whom hematopoietic stem cell transplantation is not a suitable treatment [2]. A clinical study (the electronic VELCADE^®^ OBservational Study) in a cohort of 873 patients with relapsed/refractory multiple myeloma treated with BOR after several previous treatment lines (median 2) yielded the following results [3]: (i) 69% of patients responded to treatment either partially (32%) or completely/almost completely (37%); (ii) the median time to response was 1.8 months, the median time to further treatment was 9.7 months, and the median interval without treatment was 7.9 months. The following side effects have been reported: not otherwise specified neuropathy (19%) and not otherwise specified diarrhea and thrombocytopenia (17% each). A total of 230 patients (26%) discontinued BOR treatment due to adverse events. At baseline, 689 patients (79%) had no peripheral neuropathy. After cycle 8, the incidence of peripheral neuropathy (in any grade) increased to 51% and in grade 3/4 was 12% of patients. These data characterize the efficacy and risks of treatment with relapsed/refractory multiple myeloma bortezomib [3]. BOR is a dipeptidyl boronic acid (systematic name, *N*-[(1R)-1-(dihydroxyboryl)-3-methylbutyl]-Nα-(2-pyrazinylcarbonyl)-*L*-phenylalaninamide) and is a highly specific proteasome inhibitor [4]. This inhibitory activity is the result of the boron in the BOR molecule attacking the catalytic center of the 20S core of the proteasome with high affinity [5]. Inhibition of proteasome activity results from a tetrahedral complex that forms between the hydroxyl group of a threonine residue in the 20S β5 subunit of the proteasome and a boronic acid moiety [6]. Therefore, the dominant mechanism of BOR action is the blockade of protein degradation by the proteasome, which leads to endoplasmic reticulum stress (ER stress), cell cycle (CC) arrest, and subsequent apoptosis [7]. Several lines of evidence suggest that BOR in combination with chemotherapy may be effective in attenuating acute lymphoblastic leukemia (ALL) characterized by dysfunctional B or T cell differentiation in children, especially for those who have developed multidrug resistance (MDR) [8,9]. The relationship of the efficacy of BOR treatment to the development of MDR can be deduced from the following findings: (i) BOR may be a substrate for the ABCB1 drug transporter (also known as P-glycoprotein—P-gp) but not for other transporters, such as ABCC1 or ABCG2 [10]; (ii) BOR may interfere with the expression of several proteins active in the cell to protect against chemical stress, e.g., the ABCC1 drug transporter [11] and P-gp [12]; (iii) expression of P-gp is associated with modulated expression of molecular chaperones active in the response of cells to ER stress and cellular protection against tunicamycin, a known ER stressor [13]. However, new findings indicate that inhibition of P-gp does not increase the efficacy of proteasome inhibitors in multiple myeloma cells [12] or in P-gp-positive B-cell precursor ALL cell lines [14].

Mouse L1210 cells are lymphocytic cells for which differentiation towards B cells has been histochemically demonstrated [15]. In parental L1210 cells (S cells), we induced P-gp expression either by adaptation to vincristine (vincristine-treated cells, R cells, [16]) or by transfection with a human gene encoding P-gp (transfected cells, T cells, [17]). Both P-gp-positive cell variants (R and T cells) showed significant resistance to P-gp substrates compared to S cells. In addition, the R and T cell lines showed significant resistance to substances not related to P-gp, such as cisplatin [18], tunicamycin [19,20], and thapsigargin [21]. P-glycoprotein has, in addition to drug efflux activity, modulatory effects that suppress the initiation of programmed cell death under the influence of cytotoxic substances (reviewed in [22]). Some additional characteristics of these cell variants are summarized in Table S1 in the supplementary files. We hypothesized that P-gp-mediated MDR is functionally linked to cellular responses to ER stress, which together modulate cellular sensitivity to various drugs [23]. Consistent with our hypothesis, we have shown that in P-gp-positive R and T, regulatory chaperones of the unfolded protein response (UPR), are altered, which leads to resistance against tunicamycin, an ER stressor [13]. The accumulation of unfolded proteins in the ER causes ER stress and can be achieved by treating cells with several substances (ER stressors), including tunicamycin (an *N*-glycosylation blocker), thapsigargin (an ER calcium ATPase inhibitor), BOR, and MG-132 (a proteasome inhibitor, for the chemical structure, see Figure S1 in the supplementary files) [24]. Since we showed that P-gp-positive R and T cells are resistant to thapsigargin [21] and tunicamycin [19,20], which cannot be explained by the transport activity of P-gp, we decided to further investigate the resistance of cells to ER stressors. For this purpose, we prepared variants of L1210 cells selected for resistance by all four ER stressors described above [25]. We found that the main molecular feature of these variants is the overexpression of the molecular chaperone GRP78/BiP, which is the central regulator of ER stress. Interestingly, overexpression of this chaperone was found in the R and T cells, but not the S cells, and this property appears to be critical for the resistance of R and T cells to tunicamycin [13]. In contrast to the reduced sensitivity of R and T cells to thapsigargin and tunicamycin, the response of these P-gp-positive cells to BOR was similar to that of the P-gp-negative S cells [25]. These results are consistent with the finding that MDR proteins (P-gp, multidrug resistance protein-1 and both breast and lung cancer resistance proteins) did not affect the cytotoxicity of BOR [26]. This finding is interesting because it indicates that BOR may be used alone or in combination with other antileukemic agents for the treatment of recurrent blood malignancies. Therefore, in this work, we decided to examine the effects of BOR-induced cell damage on S, R, and T cells in more detail.

## 2. Results

### 2.1. Effect of Bortezomib on the Expression and Activity of P-glycoprotein

We performed the study on three variants of L1210 cells: parental P-gp-negative S cells and two P-gp-positive R and T cells. We have studied the panel of these cells in detail in the past, and some of their characteristics important for current paper are summarized in Table S1 in the supplementary files. In both the P-gp-positive variants, in contrast to the S cells, massive expression of P-gp at the mRNA and protein levels was detectable [17]. In the P-gp-positive cells, the intracellular retention of calcein, which is a substrate of P-gp, was reduced compared to that in the S cells [20]. Consistent with this finding, the R and T cells were much less sensitive to P-gp substrates (such as VCR and doxorubicin) than S cells [22]. We continuously checked these characteristics during the experiments.

BOR inhibited S, R, and T cell proliferation with IC_50_ values of 6.0752 ± 1.7656, 4.7708 ± 0.8911, and 3.6738 ± 0.5412 (in nM), respectively (Figure S2 in the supplementary files). This result is consistent with previously published data [25]. Because there were weakly significant differences between the IC_50_ values of the S cells and the R or T cells, it can be concluded that BOR affects S, R, and T cells with approximately equal efficacy. The results observed with vincristine treatment (prototypical substrate of P-gp) differed; vincristine had a much stronger effect on the S cells than on the R or T cells (Figure S2 in supplementary files).

**Figure 1:**
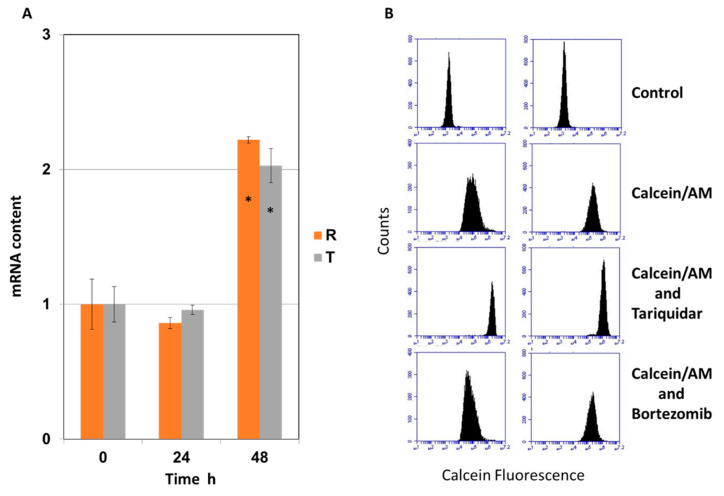
Panel (**A**): qRT-PCR detection of mRNA encoding P-gp in R and T cells cultured in medium containing BOR (5 nM) for 24 and 48 h. Experimental data represent the mean ± S_D_ of three independent experiments. Significance *—data differ from control (0) at *p* < 0.02. Panel (**B**): Detection of P-gp efflux activity by calcein/AM retention assay by fluorescence cytometry. The data are representative of three independent measurements. Tariquidar, a known inhibitor of P-gp, at a concentration of 0.5 μM, increased calcein retention in the R and T cells [29].

Culturing R and T cells for 24 h in BOR-supplemented medium (5 nM final concentration) induced a negligible decrease in P-gp transcript levels (Figure 1A). Continuation of the culture by an additional 24 h resulted in doubling of the P-gp gene transcript level in both the resistant cell variants. The expression of the MRP1 transporter (*Abcc1* gene product) in S-cells increased by 80% and in contrast in R and T cells slightly decreased (about 20%) 24 h after the addition of BOR at a concentration of 5 nM (supplementary files Figure S3 panel A). The expression of the BCRP transporter (product of the *Abcg2* gene) increases after BOR treatment in R and T cells and remains unchanged in S cells. Although these alterations may cause changes in the sensitivity of our cells to BOR, their effect appears to be small, because similar sensitivity of S, R, and T cells to BOR was detected (supplementary sets, Figure S2). This may be due to the fact that the expression of both transporters after BOR treatment did not exceed the expression of the untreated control more than twice. However, resistance mediated by either MRP1 or BCRP is often achieved by increasing their expression 10- to 100-fold [27,28].

Another question was whether BOR is able to reduce the efflux activity of P-gp. We used the calcein/AM retention assay described elsewhere [20] to measure P-gp efflux activity directly in living cells. BOR at concentrations of 1.0 and 10.0 nM only slightly altered calcein retention in the R and T cells (Figure 1B). As a control, we used the known P-gp inhibitor tariqidar (at concentration 0.5 μM), which significantly increased calcein retention.

In previous work, we have shown that tariquidar at this concentration restores calcein retention within R and T cells to the same extent as observed in S cells [29]. Another known P-gp inhibitor, verapamil, also increased calcein retention in R and T cells (not shown).

These data exclude the possibility that BOR added to R and T cells at a concentration range of 1.0–10.0 μM may significantly affect P-gp transport activity. Therefore, in additional experiments, we chose cell incubation for up to 24 h with a concentration of 5 nM BOR (which corresponds to the IC_50_ values of the S, R, and T cells at 48 h of incubation with BOR) (Figure S2 in the supplementary files). Under these conditions, we did not expect to find significant changes in the expression level of the gene for P-gp or P-gp efflux activity in the R and T cells.

### 2.2. Effect of Bortezomib on the Cell Cycling of the S, R, and T Cell Variants

**Figure 2:**
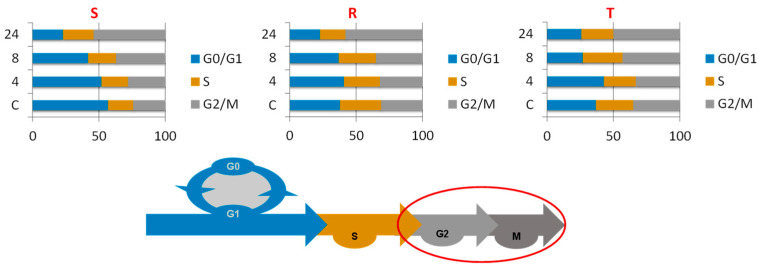
Effect of BOR (5 nM) on the cell cycle of S, R, and T cells after 4, 8, and 24 h of incubation compared to the untreated control (C). The data are representative of three independent measurements. The corresponding histograms generated from fluorescence cell cytometry data are documented in the supplementary files (Figure S4). The scheme at the bottom shows the progress of the cell cycle. The red ellipse indicates the points where the cell cycle was arrested under the influence of BOR.

In additional sets of experiments, we studied the effect of BOR (5 nM) on the transition of cells into individual phases of the CC during a 24-h passage by measuring samples obtained at 4, 8, and 24 h. We used a protocol of DNA staining with propidium iodide (PI) in cells fixed with 70% ethanol at −20 °C [30]. In the absence of BOR, the S cells differed from the R and T cells, with a larger proportion of the S cells in the G0/G1 phase (more than 50%) and a smaller proportion in the S or G2/M phase (Figure 2).

We observed a time-dependent gradual decrease in the proportion of S cells in the G0/G1 phase and a corresponding increase in the proportion of S cells in the G2/M cell phase (Figure 2). The proportion of cells in the individual phases of the CC did not change considerably in R cells passaged in the presence of BOR for 4 and 8 h or in T cells passaged in the presence of BOR for 4 h. However, the passage of R cells for 24 h and T cells for 8 and 24 h in the presence of BOR led to a decreased proportion of both these cell variants in the G0/G1 phase and an increased proportion of these cell variants in the G2/M phase.

### 2.3. Effects of Bortezomib on the Expression of Proteins Associated with CC Progression in S, R, and T Cells

**Figure 3:**
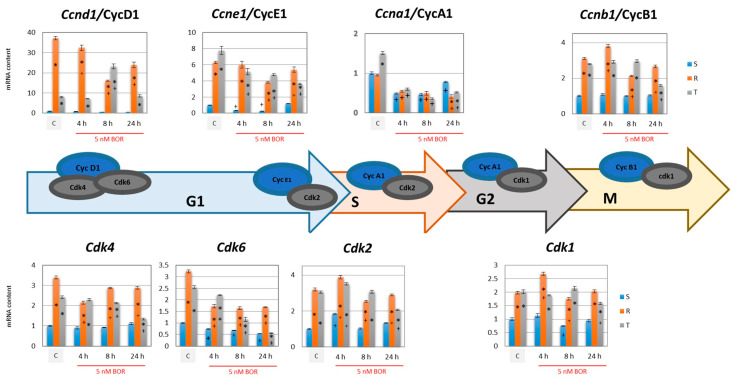
Effect of BOR (5 nM) on the expression of CDKs and their activating cyclins in S, R, and T cells. β-actin was used as an internal standard and relative quantity of mRNA for S cells in the absence of BOR (C) was arbitrarily taken as one. The results represent the means ± SD of three independent measurements. Statistical significance: *—data differ from data obtained for S cells treated equally at *p* < 0.02; +—data for individual cell variants after BOR treatment differ from the untreated control at *p* < 0.02. The scheme shows the progress of the cell cycle with checkpoints representing CDKs in complexes with activating cyclins. CC is regulated by cyclin-dependent threonine or serine kinases (CDKs encoded in mice by the *Cdk1*, *Cdk2*, *Cdk4,* and *Cdk6* genes), whose activity depends on their association with activating cyclins A1, B1, D1, and E1 (encoded in mice by the *Ccna1*, *Ccnb1*, *Ccnd1,* and *Ccne1* genes) [32]. Cyclin D1-activated CDK4 and CDK6 activities [33] and cyclin E1-activated CDK2 activity [34] are important for the progression from G0/G1 phase to S phase of CC. CDK2 and CDK1, both activated by cyclin A1, allow progression through the S and G2 phases of CC, respectively [35,36]. Cyclin B1-activated CDK1 is functional during M phase CC [36].

**Figure 4:**
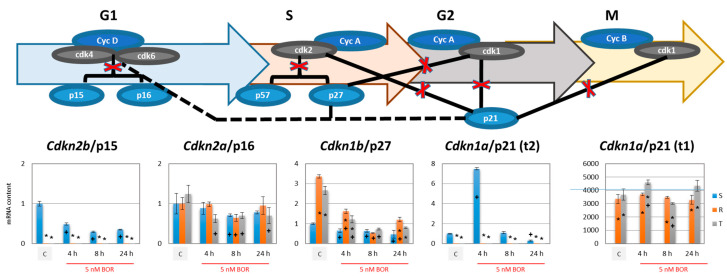
Effect of BOR (5 nM) on the expression of CDKs inhibitors (*Cdkn2b, Cdkn2a,* *Cdkn1a,* and *Cdkn1b*) in S, R, and T cells. Gene for β-actin was used as an internal standard and relative quantity of mRNA for S cells in the absence of BOR (C) was arbitrarily taken as one. The results represent the means ± S_D_ of three independent measurements. Statistical significance: *—data differ from data obtained for S cells treated equally at *p* < 0.02; +—data for individual cell variants after BOR treatment differ from those of the untreated control at *p* < 0.02. In qRT-PCR detection of p57 gene (*Cdkn1c*) expression in all three cell variants, the p15 gene expression in R and T cells, p21 transcript variant t2 in R and T cells, and p21 transcript variant t1 in S cells, the measurable signal started to emerge when the number of cycles exceeded 36, which we considered a negative result. This limit was based on the recommended optimal cutoff for qPCR [37]. The scheme shows cell cycle progression with checkpoints representing CDKs with either their activating cyclins or their inhibitors. There are two families of CDK inhibitors. INK4 proteins (p15 and p16) inhibit the kinase activity of cyclin D complexes with CDK4 and CDK6, thereby attenuating the G1/S CC transition [38]. In contrast, KIP/CIP proteins (p21, p27, and p57) inhibit the kinase activities of CDK1 complexed with cyclins A and B or CDK2 complexed with cyclin A [39]. Therefore, these proteins slow CC progression in the S and G2/M phases. Paradoxically, the p21 and p27 proteins stabilize the cyclin D complex with CDK4 and CDK6 and thus may facilitate transitioning from the G0/G1 phase to the S phase [39].

The CC consists of a series of individual events that together lead to the division of one cell into two cells. Several checkpoints (CDKs activated with cyclins) distinguish the different stages of the active CC [31]. In contrast, endogenous protein inhibitors of CDKs negatively regulate CC progression. The role of individual cyclins, CDKs, and their protein inhibitors in the CC is documented in the schemes presented in Figure 3 and Figure 4.

The expression of cyclins B1, D1, and E1 (encoded in mice by the *Ccnb1*, *Ccnd1,* and *Ccne1* genes, respectively) was significantly higher in the P-gp-positive R and T cells than in the P-gp-negative S cells (Figure 3). We also observed an increase in the expression of the *Cdk1*, *Cdk2*, *Cdk4,* and *Cdk6* genes in the R and T cells compared with the S cells. Increased expression of CDKs and their activating cyclins is probably critical for the accelerated proliferation of the R and T cells compared to the S cells, which has been previously described [25]. Increased expression of the genes encoding CDK4 and CDK6 and their activator, cyclin D1, as well as CDK2, with activating cyclin E1 in P-gp-positive R and T cells (Figure 3), accelerates the shift of CC from the G0/G1 to S phase. This difference in CC expression explains why the proportions of R and T cells in the GO/G1 CC phase were relatively lower than the proportion of S cells (Figure 2). Elevated expression of the *Cdk2* and *Cdk1* genes in R and T cells allowed them to pass more rapidly through the S and G2 phases, although there was no significant difference in the expression of the *Ccna1* gene (encoding cyclin A1), which activated both CDKs in these phases. In the M phase, CDK1 is activated by cyclin B1, whose genes were also more highly expressed in the R and T cells than in the S cells (Figure 3). Thus, it can be concluded that the differences in the CC progression between the P-gp-negative S cells and P-gp-positive R and T cells are due to specific differences in the expression of CDKs and their cyclin activators.

The expression of CDK genes under the influence of BOR oscillates around the values found for untreated cells (Figure 3). Over time, we found a monotonic decrease in expression only for *Cdk6* for all three cell variants. Slight changes in *Ccnb1*, *Ccnd1,* and *Ccne1* expression were also visible under the influence of BOR, which was more visible in R and T cells than in S cells. In contrast, *Ccna1* expression was downregulated in all three cell variants after BOR treatment. This downregulation is at least partly responsible for the arrest of cells in the G2/M phase.

CDK activity is negatively controlled by inhibitors of two gene families [31,40]. The first are INK4 (inhibitors CDK4 and 6) proteins (p15 and p16, also known as INK4B and INK4A, encoded in mice by *Cdkn2b* and *Cdkn2a* genes), which inhibit the kinase activity of cyclin D complexes with CDK4 and CDK6 (Figure 4), thereby attenuating the G1-S CC transition [38]. Second are CIP/KIP (CDK-interacting proteins/kinase-blocking proteins) proteins (p21, p27, and p57, also known as CIP1, KIP1, and KIP2, encoded in mouse by *Cdkn1a*, *Cdkn1b,* and *Cdkn1c* genes), which inhibit the kinase activities of CDK1 complexed with cyclin A1 and B1 or CDK2 complexed with cyclin A1 [39]. Therefore, these proteins slow CC progression in the S and G2/M phases (Figure 4).

While the p16 protein gene (*Cdkn2b*) was similarly expressed in all three variants of L1210 cells, the p15 protein (*Cdkn2a*) was expressed only in S cells (Figure 4). Thus, it can be stated that the abandonment of the G0/G1 phase is suppressed in S cells by both inhibitors of CDK4 and CDK6 acting there and in R and T cells by only one. This, in turn, contributes to the understanding of why the proportion of R and T cells in the G0/G1 phase of CC is relatively lower than that in S cells. The expression of the *Cdkn2a* gene (encoding p16) in S, R, and T cells or the expression of the *Cdkn2b* gene (encoding p15) in S cells decreased after BOR treatment depending on the duration of action (Figure 4).

**Figure 5:**
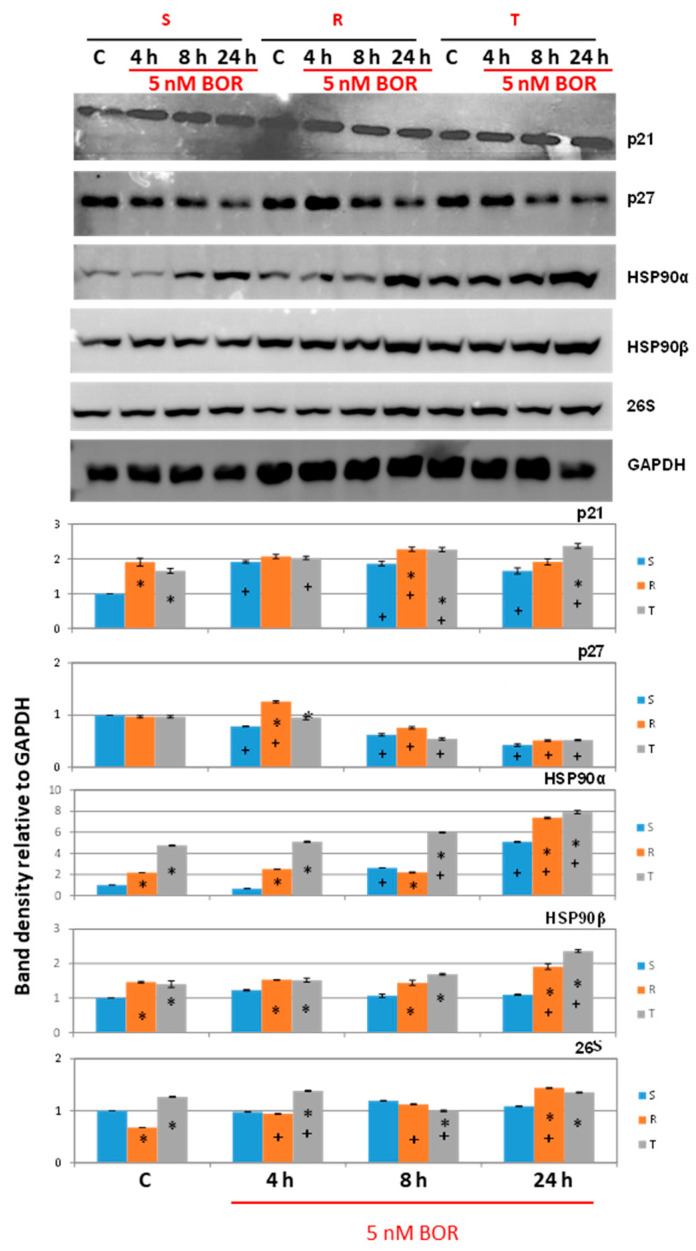
Levels of p21, p27, HSP90α, HSP90β, and 26S proteasome in S, R, and T cell variants as determined by Western blot analysis. The cells were incubated for 4, 8, and 24 h in growth medium in the absence (C) or presence of BOR (5 nM) in a CO_2_ incubator. GAPDH was used as an internal control. The data are representative of three independent measurements. The optical densities of the protein bands were quantified by densitometry and then expressed relative to GAPDH and summarized in bar graphs. The data represent the mean ± S_D_ of three independent measurements. Statistical significance: *—data differ from data obtained for S cells treated equally at *p* < 0.02; +—data for individual cell variants after BOR treatment differ from the untreated control at *p* < 0.02.

We did not find a measurable number of transcripts for the p57 protein in any of the three cell variants (not shown). This finding was independent of BOR treatment. In contrast, p27 expression was observed in all three cell variants, being higher in the P-gp-positive variants (R and T cells) than in the P-gp-negative variant (S cells). BOR treatment suppressed the expression of this protein (Figure 4). Similar behavior was found for the p27 protein by Western blot analysis (Figure 5).

We obtained the most interesting results in the expression of p21. The p21 protein can be expressed from two different mRNA transcript variants, which encode the same full-length protein [41]. This finding is documented in Figure S5 (supplementary files). In S cells, the p21 protein was expressed from a common transcript variant (transcript t2; Figure S5 in the supplementary data), and in R and T cells, the mRNA for this variant did not exist. However, the P-gp-positive cells contained an alternative, the t1 transcript (Figure S5 in the supplementary data). The entire p21 protein was then detectable in both the P-gp-negative S cells (in small amounts) and P-gp-positive R and T cells (in high amounts) by Western blot analysis (Figure 5). Incubation of the S cells with BOR for 4 h induced a massive increase in the level of t2 transcript, which was reduced to the control level upon extension of the incubation to 8 and 24 h (Figure 4). Accordingly, we detected an increase in p21 protein levels in BOR-treated S cells by Western blotting for four hours, and this increase persisted for 8 and 24 h (Figure 5). Fluctuating levels of t1 transcripts around the control value (in the absence of BOR) were detected in the R and T cells after BOR treatment (Figure 4). The p21 protein levels in the R and T cells tended to increase slightly (in some cases significantly) after treatment of the R and T cells with BOR (Figure 5).

### 2.4. Effects of Bortezomib on the Quantity and Linkage of Ubiquitination

As mentioned above, BOR blocks the degradation of misfolded proteins by inhibiting the activity of the 20S subunit of the proteasome. Proteins labeled with polyubiquitin chains undergo proteasomal degradation [42].

**Figure 6:**
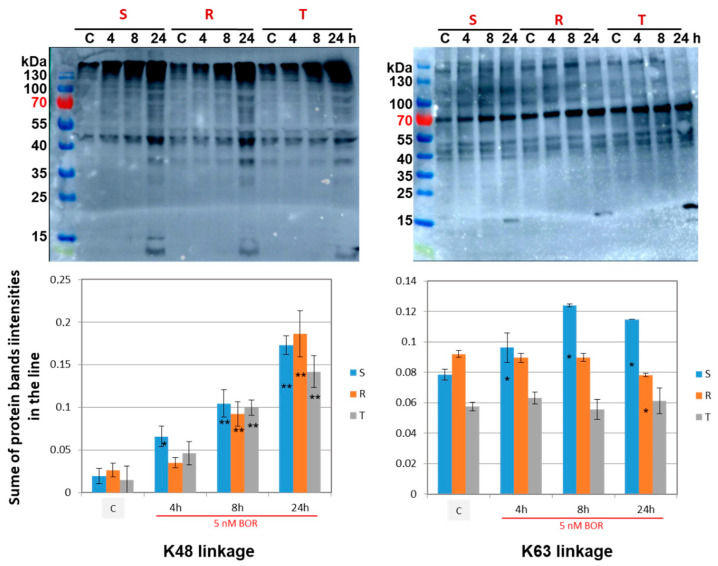
The levels of protein ubiquitination through K48 or K63 linkage in S, R, and T cell variants were determined by Western blot analysis using K48- and K63-linkage specific antibodies. Cells were incubated for 4, 8, and 24 h in growth medium in the presence of BOR (5 nM) in an incubator with 5% CO_2_ in air and were compared with untreated cells (C). The correct dosing of proteins into the individual lanes was verified in SDS-PAGE gels after staining with Coomassie G-250 (not shown). The data are representative of three independent measurements. The optical densities of all protein bands in separate lanes were quantified by densitometry and are expressed relative to the total optical density of all the bands in the entire blot. The data are summarized in bar graphs. The data are expressed as the mean ± S_D_ of three independent measurements. Statistical significance: the data for individual cell variants after BOR treatment differed from the untreated control at *p* < 0.02 (*) and *p* < 0.01 (**).

Ubiquitin chains are formed by the attachment a C-terminal carboxyl group of an added ubiquitin to the ε-amino group of the lysine in the ubiquitin molecule previously added to the chain. Because the ubiquitin molecule contains seven different lysine residues, different linkages of ubiquitin are possible. While the best-known K48 linkage is accepted as a protein marker for proteasomal degradation, the K63 linkage, which is equally abundant, is a signal for DNA repair, endocytosis, and further processing of endocytic material [43]. Therefore, we further investigated the effect of BOR on the extent of ubiquitination of proteins with K48 and K63 linkages using ubiquitin linkage-specific antibodies. The results are presented in Figure 6.

Incubation of S, R, and T cells in the presence of BOR led to a time-dependent increase in the proteins ubiquitinated by K48-linked chain addition. In contrast, only a slight (although significant) increase in protein ubiquitination via K63 linkage was present, and only in the S cells, after 8 and 24 h of incubation with BOR. In the R and T cells, incubation in the presence of BOR did not change the levels of ubiquitinated proteins with this linkage.

These results demonstrate an increase in proteins ubiquitinated via K48 linkage (substrates of proteasomal degradation [42,43]) after inhibition of proteasome activity by BOR. In contrast, the levels of proteins that were ubiquitinated through the K63 linkage (which does not predispose the proteins to proteasomal degradation [42,43]) remained practically unchanged under conditions of proteasome inhibition by BOR.

### 2.5. Effects of Bortezomib on the Expression of Deubiquitinating Enzymes

**Figure 7:**
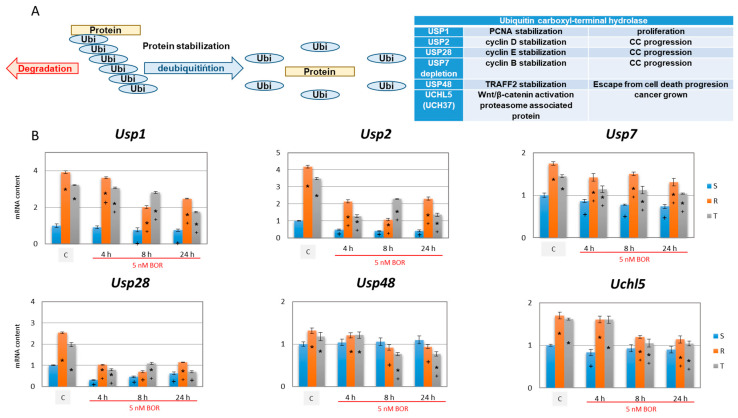
Effect of BOR (5 nM) on the expression of deubiquitinase genes in S, R, and T cells. Panel (**A**): The role of some deubiquitinases in the survival and life cycle of neoplastic cells: (i) Ubiquitin-specific protease 1 (USP1) prevents lethal stimulus, i.e., the persistence of monoubiquitinated proliferating cell nuclear antigen (PNCA) on the replication fork [44]; (ii) USP2 and USP28 stabilize cyclin D [45] and cyclin E [46], respectively; (iii) depletion of USP7 alters mitotic progression and stabilizes cyclin B [47]; USP48 stabilizes TRAF2, which acts against cell death [48,49]; and finally, UCHL5 (ubiquitin C-terminal hydrolase L5, also known as UCH37) accelerates cancer growth through upregulation of the Wnt/β-catenin signaling pathway [50], which is a proteasome-associating protein [51]. Panel (**B**): qRT-PCR detection of USP1, USP2, USP7, USP28, USP48, and UCHL5 transcripts. β-actin was used as an internal standard and relative quantity of mRNA for S cells in the absence of BOR (C) was arbitrarily taken as one. The results represent the means ± S_D_ of three independent measurements. Statistical significance: *—data differ from the data obtained for S cells treated equally at *p* < 0.02; +—data for individual cell variants after BOR treatment differ from those of the untreated control at *p* < 0.02.

In further experiments, we investigated the effect of incubating S, R, and T cells in medium with 5 nM BOR for 4, 8, and 24 h on expression of deubiquitinases. The results are shown in Figure 7.

Expression of deubiquitinases *Usp1*, *Usp2*, *Usp7*, *Usp28*, and *Uchl5* (for explanation of the symbolism see Figure 7) was more pronounced in the P-gp-positive R and T cells than in the P-gp-negative S cells (Figure 7). A slight, although significant, increase in *Usp48* expression was observed in the R cells compared to the S cells. The increase in the latter enzyme in the T cells compared to the level in the S cells was insignificant. Incubation with BOR induced a decrease in the expression of deubiquitinases (in several cases, significantly) in the S, R, and T cells (Figure 7). This decrease was most evident in *Usp2* and *Usp28*, which encode protein stabilizing cyclins D and E, with decreases to 50% of the values found in cells without BOR treatment. *Usp7* expression decreased less significantly under the influence of BOR in the S, R, and T cells. We did not observe a decrease in the expression of *Usp1*, *Usp48,* or *Uchl5* after BOR treatment in the S cells, but slight decreases were found in the R and T cells.

### 2.6. Effect of Bortezomib on the Expression of Proteins Required for Proteasome Formation

**Figure 8:**
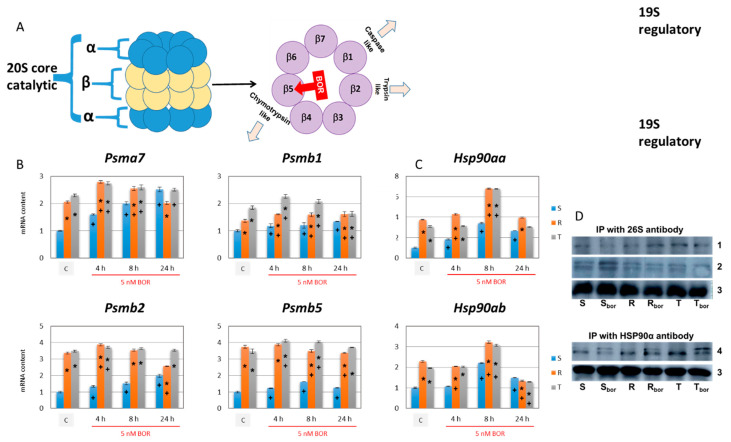
The expression of genes required for proteasome formation. Panel (**A**): Scheme of the structural organization of the proteasome. The structure of the 26S proteasome consists of the following functional parts: a proteolytic core (20S) and a regulatory cap (19S) (reviewed in [52]). The 20S core consists of pairs of fourteen proteins encoded by 14 different genes and arranged in four heptamer rings. The outer rings contain seven α subunits that form a gateway for the entry of substrates into the proteolytic site, i.e., two inner rings, each formed by seven β subunits. Three of these seven inner subunits, β1, β2, and β5, are proteolytically active (with caspase-like, trypsin-like, and chymotrypsin-like activities), the latter being inhibited by BOR. This architecture creates an internal compartment where proteolytic sites are located. Therefore, the 20S subunit degrades only proteins that have entered this microspace (reviewed in [52]). Attaching the 19S cap to one or both ends of the 20S forms a complete 26S proteasome. 19S particles recognize polyubiquitin-tagged substrates that are then degraded. Through 19S, these proteins are first deubiquitinated and then transferred to the interior of 20S, where they are cleaved into oligopeptides. The 26S proteasome is stabilized by the HSP90 chaperone associated directly with the 20S core, affecting 20S activity [53,54]. The HSP90 chaperone has ATPase activity and facilitates the attachment of 19S caps to the 20S core. In HSP90 deficiency, the free 20S subunits become prevalent (reviewed in [55]). Panel (**B**): Gene expression of *Psma7, Pmsb1, Pmsb2,* and *Pmsb5* (encoding α7, β1, β2, and β5 proteasomal subunits, respectively) in S, R, and T cells in relation to that upon incubation with BOR. Panel (**C**): Gene expression of *Hsp90aa* and *Hsp90ab* (encoding HSP90α and HSP90β, respectively) in S, R, and T cells in relation to incubation with BOR. qRT-PCR was used for detection, and the results are shown in panels B and C, and β-actin was used as an internal standard. Relative quantity of mRNA for S cells in the absence of BOR (C) was arbitrarily taken as one. Cells were incubated with 5 nM BOR for 4, 8, and 24 h before measurement, and then, total RNA was isolated. The results represent the means ± S_D_ of three independent measurements. Statistical significance: *—data differ from the data obtained for S cells treated equally at *p* < 0.02; +—data for individual cell variants after BOR treatment differ from the untreated control at *p* < 0.02. Panel (**D**): Detection of the complex formed by the 26S proteasome and HSP90 by immunoprecipitation. Whole-cell lysates were immunoprecipitated with either anti-26S or anti-HSP90α antibodies. Immunoprecipitates were subjected to Western blotting with (i) antibodies against HSP90α or HSP90β in the case of immunoprecipitation with the anti-26S antibody and (ii) antibody against the 26S proteasome in the case of immunoprecipitation with the anti-HSP90α antibody. Protein bands were visualized with 1–anti-HSP90α antibody, 2–anti-HSP90β antibody, 3–antibody against rabbit IgG, and 4–anti-26S antibody. The rabbit IgG light chain (25 kDa) was included as an internal standard of signaling. Lines: cells prior to immunoprecipitation were incubated in the absence (S, R, and T cells) or presence (5 nM) of BOR for 24 h (S_bor_, R_bor_, and T_bor_ cells). The data are representative of three independent measurements.

A functional 26S proteasome consists of a catalytic 20S core containing two inner heptamer rings with β1–β7 subunits and two outer heptamer rings with α1–α7 subunits as well as of a 19S regulatory cap composed of regulatory eubiquitination system [48], which is shown in Figure 7A. We further measured the expression levels of three β subunits (β1, β2, and β5, encoded by the *Psmb1*, *Psmb2*, and *Psmb5* genes) of the inner heptamer rings of the 20S core of the proteasome, which have proteolytic activity. As a control, we used the *Psma7* gene to express the α7 protein, which is part of the outer heptamer rings of the 20S core (see proteasome structure in Figure 8A). The expression of the *Pmsb5* gene, which encodes the β5 subunit (i.e., the target of BOR), was significantly higher in the P-gp-positive R and T cells than in their P-gp-negative S counterparts (Figure 8B). We found similar behavior for β2, encoded by the *Pmsb2* gene. A significant increase in expression was observed for the *Pmsb1* gene (encoding β1), but the increase was less pronounced than that of the *Pmsb5* and *Pmsb2* genes (Figure 8B). Under the influence of BOR, depending on the time of its action, slight changes (and some significant) in the expression of all three genes were observed. The *Pmsa7* gene encoding the α7 protein was also more highly expressed in the R and T cells than in the S cells (Figure 8B). This finding, together with consistent data on the expression of genes encoding the β1, β2, and β5 subunits, indicates increased production of the entire 26S proteasome in P-gp-positive cells. Under the influence of BOR, the expression of the *Pmsa7* gene increases monotonically with time in the S cells, but in the R and T cells, similar continuous growth was not evident.

The attachment of the 19S regulatory cap to the 20S proteasome core (Figure 8A) is facilitated by the molecular chaperone HSP90, in the heat shock protein family [55]. The two genes *Hsp90aa* and *Hsp90ab* encode inducible HSP90α and constitutive HSP90β, respectively [56]. Therefore, we measured the expression level of both genes in relation to BOR treatment. A higher level of expression of both genes was present in the P-gp-positive R and T cells than in the P-gp-negative S cells. Accordingly, we also found increased levels of HSP90α and HSP90β proteins in the R and T cells than we found in the S cells (Figure 5). BOR treatment caused an increase in the expression of the *Hsp90aa* gene after 4, 8, and 24 h and the *Hsp90ab* gene after 8 and 24 h in the S cells compared to the untreated control cells. An increase in *Hsp90aa* expression was observed in the BOR-treated R cells after 4 and 8 h and in the BOR-treated T cells after 8 h. *Hsp90ab* is overexpressed in the R and T cells 8 h after BOR administration, and its expression paradoxically decreases below the control level after 24 h. The increase in HSP90α and HSP90β protein levels after BOR addition detected with isoform-specific antibodies is documented in Figure 5, which shows that it was particularly pronounced after 24 h. In parallel sets of experiments, we used an antibody that reacted with both HSP90 isoforms and obtained confirmatory results through Western blotting and immunocytochemistry with confocal microscopy (Supplementary files, Figure S6).

We also measured 26S proteasome levels in S, R, and T cells using an anti-26S proteasome-specific antibody, which led to the identification of a M_r_~100 kDa protein in a band in the Western blot. The level of the 26S proteasome in the BOR-treated and BOR-untreated cells oscillated at approximately one (Figure 5), which was arbitrarily chosen and represents the optical density of the 26S protein band in untreated S-cells. The formation of the 26S proteasome is stabilized by HSP90, which is associated with the 20S proteasome core [53,54]. Therefore, we detected an association between the 26S proteasome and HSP90α or HSP90β proteins by immunoprecipitation with anti-26S proteasome antibody and anti-HSP90α antibody. The immunoprecipitate obtained using the anti-26S proteasome antibody was subjected to Western blotting with the anti-HSP90α and anti-HSP90β antibodies, and then, the immunoprecipitate obtained with the anti-HSP90α antibody was subjected to Western blotting with the anti-26S proteasome antibody. The results are summarized in Figure 8D. There was an observable trend of increasing immunoreactivity of the HSP90α antibody and, in contrast, decreasing immunoreactivity of the anti-HSP90β antibody in immunoprecipitates obtained with the anti-26S proteasome antibody isolated from the R and T cells compared to the S cells. In immunoprecipitates obtained with the anti-HSP90α antibody, the immunoreactivity of the anti-26S proteasome antibody was increased in the R and T cells compared to the S cells. Western blot detection of proteins obtained from immunoprecipitation formed doublets in some cases, which may be related to differences in posttranslational modifications of these proteins (glycosylation, ubiquitination, etc.).

### 2.7. Measurement of Proteasome Activity in S, R, and T Cells

We further focused on monitoring proteasome activity in cell homogenates obtained from S, R, and T cells. We resolved the problem caused by incubating cells in medium with 5 nM BOR for 4 h or by incubating cells in medium with a known inhibitor of HSP90 function, a geldanamycin derivative [57], AADG (17-(allylamino)-17-demethoxygeldanamycin at a final concentration 1 μM, for the structure see Figure S1 in supplementary files) for 24 h. After incubation, the cells were harvested by centrifugation, washed extensively in phosphate-buffered saline (PBS) to remove inhibitors, and then used to measure proteasome activity. An ab107921 proteasome activity assay kit (Abcam, Cambridge, UK) was used for the measurement according to the manufacturer’s instructions [58]. The specificity of the test was ensured by (i) adding a specific substrate of the 26S proteasome, Suc-LLVY-AMC (for the structure, see Figure S1 in the supplementary files) and (ii) adding specific 26S proteasome inhibitor MG-132 (for the structure, see Figure S1 in supplementary files). The substrate was labeled with fluorescently active 7-amino-4-methylcoumarin (AMC), which is released by chymotrypsin-like proteasome activity. MG-132 was used to distinguish other proteolytic activities that can lead to similar cleavage of the substrate.

**Figure 9:**
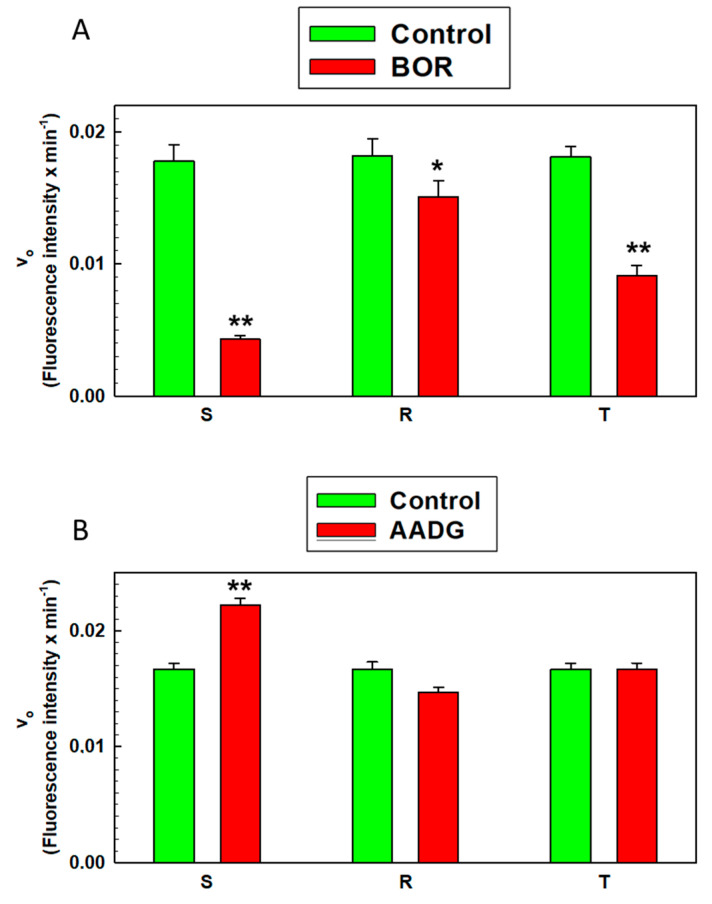
Proteasome activity was measured by an ab107921 proteasome activity assay kit. Panels (**A**,**B**) represent measurements of the 26S proteasome activity in S, R, and T cells after incubation with either 5 nM BOR (for the structure, see Figure S1 in the supplementary files) for 4 h or 1 μM AADG (for the structure, see Figure S1 in the supplementary files) for 24 h. The data represent the computed values ± SD as determined accounted from the linear regression results shown in Figure S7 (in the supplementary files) for three independent measurements. Significance: the value differed from the control (untreated cells) at **—*p* < 0.01 or *—*p* < 0.02.

Cutting AMC from the substrate and plotting the BOR effect as a function of time for 90 min revealed a straight line (Figure S7A in the Supplementary files), and similarly, a straight line was obtained when plotting the AADG effect measured for 75 min (Figure S7B in the supplementary files), both indicating a zero-order reaction that is typical of enzyme catalysis. In previous work, we observed similar behavior when measuring proteasome activity in L1210 cell variants [25]. This behavior makes it possible to determine the initial reaction rate of the proteasomal cleavage of a substrate, which is constant throughout the measurement, as the slope of the respective line. The values of the initial rates of proteasome activity are documented in Figure 9.

The proteasome activity in the homogenates obtained from S, R, and T cells reached nearly the same level (Figure 9). Four hours of incubation of the S cells with 5 nM BOR caused a decrease in 26S proteasome activity to approximately 25% of the baseline (Figure 9A). In contrast, the effect of BOR was significantly weaker on the R cells, which after incubation with BOR exhibited approximately 83% of the original activity. The value of the decrease in proteasome activity induced by BOR in the T cells was between the values obtained with the S and R cells and reached approximately 50% of the original value (Figure 9A).

Incubation of S cells with 1 μM AADG caused an increase in 26S proteasome activity, to 133% of the original activity (Figure 9B). Under the same conditions, the 26S proteasome activity in R and T cell homogenates changed very little (approximately 88% for the R cells and approximately 100% for the T cells) compared to controls cultured in the absence of AADG (Figure 9B).

## 3. Discussion

BOR, a proteasome inhibitor, is a progressive therapeutic agent currently used clinically to treat multiple myeloma and mantle cell lymphoma as indicated by their FDA and EMA approval [1,2]. Clinical/experimental studies have shown that BOR alone or in combination with other drugs may be effective in better management of other types of human hematological malignancies (reviewed in [4]).

However, neoplastic cells in response to previous treatment or due to the nature of the tissue from which they were transformed may have activated mechanisms that protect them from cell death induced by various therapeutics. There are a wide variety of mechanisms that allow cells to cope with toxic substances [22,59]. The expression of drug transporters in the ABC gene family, of which P-gp is the best known and the most common [22], is the predominant cause of chemotherapy failure in malignancy.

Therefore, we sought to determine whether P-gp-overexpressing cells respond to BOR with a similar or different intensity as their P-gp-negative counterparts. In this respect, it was interesting that the effect of BOR on the P-gp-positive R and T cells was even more pronounced than that on the P-gp-negative S cells (Figure S2 in the supplementary files). BOR is considered a P-glycoprotein substrate [10], which was expected to result in reduced BOR sensitivity of the R and T cells compared to the S cells, which was not confirmed by the data, as shown in Figure S2 (in the supplementary files). However, MDR transporters do not affect BOR cytotoxicity [26]. Measurements of P-gp transport activity by calcein/AM assay did not show elevated calcein retention of either the R or T cells in the presence of BOR. Both of these findings suggest that BOR used at these concentrations to treat R and T cells was not removed from the cells by P-gp and was not efficient enough to affect P-gp-mediated transport. Therefore, it is unlikely that BOR is a suitable substrate for P-gp expressed from the mouse genome in R and cells or from a plasmid with the human gene in T cells.

We previously described several differences between P-gp-positive R and T cells and their P-gp-negative S counterparts, which, in conjunction with or independent of P-gp efflux activity, modulated changes in the overall sensitivity of the R and T cells to different substances [13,18,19,20,21,22,25]. These changes may include a lower proportion of R and T cells than S cells in the G0/G1 phase of the cell cycle when none of the three are treated with BOR (Figure 2). This may be due to higher expression levels of genes for the major regulators of G1 phase exit in the R and T cells than in the S cells, namely, CDK4 and CDK6, which are both activated by cyclin D1 [33], and CDK2, which is activated by cyclin E1 [34], as documented in Figure 3. The faster exit from the G1 phase of the cell cycle was also associated with very low expression of the p15 inhibitor of CDK4 and CDK6 [38] in the R and T cells compared to the S cells (Figure 4).

Another reason that R and T cells exit the G1 phase rapidly relates to the higher content of the p21 protein in these cells (Figure 5). In addition to being an inhibitor of CDK2 and CDK1 when in complex with cyclin A1 or CDK1 when in complex with cyclin B1 (both cyclins are active in the S, G2, and M phases [39], see scheme on Figure 4), p21 also regulates G1 phase events. Cyclin D1 and p21 have been shown to stabilize each other through the formation of the cyclin D1-CDK4 (6)-p21 complex, and the uncomplexed proteins are readily degraded [39,60].

Although S, R, and T cells show similar sensitivity to BOR (IC_50_ values in the range of 4.3–7.8, 3.9–5.7, and 3.1–4.2 nM for the S, R, and T cells, respectively), a more detailed analysis of the cellular response features during CC progression after BOR treatment revealed several differences between these variants:

1. S cells showed a greater tendency to enter CC arrest in the G2/M phase than R or T cells, which manifests as S cells arrested within 4 h of treatment (Figure 2).
2. The gene expression of cyclins B1, D1, and E1 and all four CDKs was higher in the R and T cells than in the S cells (Figure 4). This expression level was maintained for CDK1 and CDK2 (active in S and G2/M phase [35,36]) and cyclins B1 (activating CDK1 in the M phase [36]), D1, and E1 (both activating CDK4 and CDK6 in the G1 phase [33,34]) even 24 h after the addition of 5 nM BOR. In the T cells but not in the R cells, 24 h after the addition of BOR, the expression levels of the CDK4- and CDK6-encoding genes approached the levels in the S cells. Cyclin A1 (activating CDK2 in the S phase [35] and CDK1 in the G2 phase [36]) gene expression levels decreased after treatment of all three cell variants with BOR (Figure 4).
3. *Cdkn2b* (encoding the p15 inhibitors CDK4 and CDK6 [38]) was expressed at a lower level in the R and T cells than in the S cells and persisted at this level even after BOR treatment, although the expression of this gene in the S cells decreased (Figure 4). Higher expression levels of the *Cdkn1b* gene (the p27 inhibitor CDK1 and CDK2 and stabilizer of the CDK4 and CDK6 complexes formed with cyclin D1 [39]) was observed in the R and T cells than in the S cells, a trend that persisted even after BOR treatment, although the gene expression levels for this protein decreased in all three cell variants (Figure 4). Consistent with its gene expression, the p27 protein level also decreased in all three cell variants after treatment with BOR (Figure 5). Expression of a multimodal modulator of CDKs encoded by the *Cdkn1a* gene (p21, an inhibitor of CDK1 and CDK2 and stabilizer of complexes formed by CDK4 or CDK6 with cyclin D1 [39,60]) is expressed from a common t2 transcript variant in S cells and from an alternative t1 transcript variant in R and T cells (Figure S5 in supplementary files).

It can be emphasized that, although the regulation of the course of the CC is very complex, the changes in CDK, cyclin, and CDK inhibitor expression described above correspond to differences in the CC in S, R, and T cells and their specific responses and BOR therapy.

The reason that p21 is expressed from the alternate t1 transcript in the P-gp-positive R and T cells has not been fully elucidated. However, this result may be related to the expression and function of p53 in these cells. We measured a set of experimental data in which the expression of the p53 protein was decreased in the R and T cells but not in the S cells, similar to previous findings showing that the presence of this protein appeared to be reduced in both P-gp-positive cell variants [61]. While the expression of p21 that is encoded in the common t2 transcript is strictly dependent on the presence of p53 [62], the expression of p21 that is encoded in the alternative transcript t1 is also apparent in the absence of p53 [41]. Although it is generally recognized that p21 expression is commonly induced by p53, p53-independent induction of p21 in neoplastic cells has been found in the regulatory mechanisms of cells and may be associated with genomic instability caused by the deregulation of the replication process in cancer cells [63]. The expression of p53 in S, R, and T cells and its changes in response to treatment with BOR and other inducers of ER stress are currently being intensively studied in our laboratory, and preliminary results confirm the differences between P-gp-positive R and T cells and their P-gp-negative S cell counterparts. p21 is evidently encoded in multiple transcriptional variants in both mice [64] and humans [41]. Despite the differences in the variants, the coding sequences are identical and therefore produce the same protein [65]. Transcript variant differences are based on different 5′-untranslated regions. These regions play crucial roles in regulating translation, which suggests that the expression of p21 that is encoded these variants is controlled differently at the translation level. It has been shown that 5′-upstream open reading frames in the 5′-untranslated region of gene transcription variants can increase translation during amino acid deprivation-induced stress [65]. It can therefore be assumed that under stress conditions, genes may be expressed through a different transcript variant than that transcribed in unstressed cells. Therefore, we speculate that the expression of the t1 transcript in the R and T cells is due to the response of these P-gp-positive cells to stress induced by cytotoxic substances.

Suppressed degradation of ubiquitinated unfolded proteins in BOR-inhibited proteasomes results in the accumulation of these proteins in the cells. This has been demonstrated, e.g., in BH4 acute promyelocytic leukemia cells [66]. In our experiments, we observed an increase in ubiquitinated proteins with specific K48 linkage of the ubiquitin chain (Figure 6). Polyubiquitination through this type of linkage is recognized by the 26S proteasome, and the labeled protein undergoes subsequent degradation [67]. In contrast to the K48 linkage, the polyubiquitination of proteins via the K63 chain, which is a signal for endocytosis and DNA repair [43], was increased slightly only in the S cells and appeared to be independent of BOR treatment in the R and T cells (Figure 6).

Protein ubiquitination is a dynamic, precisely controlled, and reversible process. While ubiquitination is a modification realized by the cooperation of three types of enzymes, ubiquitin activating enzymes, ubiquitin conjugating enzymes, and ubiquitin ligating enzymes, the reversal of ubiquitination of client proteins is ensured by deubiquitinases [68]. Deubiquitination can considerably contribute to the stabilization of proteins, promoting the proliferation and aggressive characteristic of neoplastically transformed cells. Some of these deubiquitinases are directly involved in the mechanism of survival and proliferation of neoplastic cells (see Figure 7). Higher expression of deubiquitinase genes (Usp1, Usp2, Usp7, Usp28, Usp48, and Uch15) was observed in the P-gp-positive R and T cells than in the S cells (Figure 7). This outcome was most pronounced for the genes for Usp1, Usp2, and Usp28. The deubiquitinase USP1 protects against response to the death signal, which causes persistence of monoubiquitinated PNCA on the replication fork. In the absence of USP1, persistent monoubiquitination of PCNA on the replication fork results in cell death [44]. Therefore, USP1 facilitates DNA replication in cells, which enables their proliferation. USP2 and USP28 stabilize cyclin D1 [45] and E1 [46], both of which are required for the activation of CDKs in the G1 phase of the CC (see scheme in Figure 3). It is therefore not surprising that the deubiquitinase genes USP1, USP2, and USP28 are more highly expressed in R and T cells, which proliferate faster [25] and remain expressed at lower levels in S cells, which are more retained in G0/G1 phase (Figure 2). The expression of deubiquitinase genes tended to decrease after BOR treatment (Figure 7). This outcome may then contribute to the increase in ubiquitinated proteins with a K48 linkage after BOR treatment (Figure 6), which are degraded to a lesser degree by the 26S proteasome and are less extensively deubiquitinated.

The proteasome (26S) is a complicated multiprotein complex that degrades poorly assembled proteins previously labeled with ubiquitin [69,70]. It consists of a catalytic subunit, 20S, and a control subunit, 19S (reviewed in [52]). The structure of the proteasome is shown in the scheme in Figure 8A. The subunits of the 20S catalytic core, β1, β2, β5 (components of inner heptamers), and the α7 (component of outer heptamers) were more highly expressed in the P-gp-positive R and T cells than in the P-gp-negative S cells (Figure 8B). Incubation of the S, R, and T cells with BOR led to slight increases in the expression of *Psma7*, *Psmb1*, *Psmb2*, and *Pmsb5* genes encoding the α7, β1, β2, and β5 subunits of the 26S proteasome, respectively (in several cases, the expression differences were significant). However, these changes do not appear to be sufficient to change the overall proteasome activity in the cells to the extent that the cellular response to BOR is altered. Consistent with this assumption, Western blot analysis revealed only small, albeit in some cases significant, changes in the expression of proteins with immunoreactivity to the 26S antibody (Figure 5).

HSP90α and β chaperones are key regulators of various processes in eukaryotic cells that facilitate client protein formation into common intermolecular complexes with a specific biological role, such as the 26S proteasome [71]. HSP90 is conjugated to the 20S core of the proteasome and facilitates the attachment of 19S regulatory caps, thereby completing the 26S proteasome complex [55]. In agreement with the roles of these chaperones, we performed immunoprecipitation assays and demonstrated a direct interaction between the 26S proteasome and HSP90α or HSP90β (Figure 8D). The function of the 19S structure is to recognize polyubiquitinated proteins with the K48 linkage and move them into the 20S core for degradation [51]. If the 19S cap is not attached, the 20S core alone is capable of splicing proteins, but in a ubiquitin-independent manner [52]. The expression of the HSP90 chaperone, which exists in mice as either inducible HSP90α (encoded by the *Hsp90aa* gene) or constitutive HSP90β (encoded by the *Hsp90ab* gene) [72], is higher in P-gp-positive cells at both the mRNA (Figure 8) and protein levels (Figure 5, Figure S6 in supplementary files). In our cell experiments, incubation with BOR for 8 h caused an increase in the transcripts of both HSP90 genes (Figure 8). After 24 h, the level of these transcripts was close to that of the unaffected controls. Incubation of the cells in the presence of BOR led to an increase in the HSP90α protein content in all three L1210 cell lines and HSP90β in the P-gp-positive R and T cells (Figure 5).

The data presented in the previous paragraph suggest that subunits for the construction of the 26S proteasome are expressed in S, R, and T cells and that the chaperone HSP90, which is necessary for the attachment of 19S caps to the 20S core, is also conjugated to the 26S proteasome (Figure 5 and Figure 8). Next, we verified the functionality of the proteasome. Specifically, we determined the proteolytic activity of the 26S proteasome. Consistent with previous work [25], measurement of proteasome activity in cell homogenates provided a linear time course, indicating a zero-order reaction (Figure S7 in the supplementary files). S cells incubated with BOR showed reduced proteasome activity to one-quarter of the value of the untreated control cells (Figure 9A). In the R and T cells, the decrease was less extensive, and the activity in these cells after incubation with BOR reached approximately 83% and 50% of the activity in the untreated control cells. This result may be related to the higher levels of expression of the proteasome α and β subunits and HSP90 chaperone in the P-gp-positive cells than in the P-gp-negative cells (Figure 5 and Figure 8). We also used the geldanamycin derivative AADG, which is a known inhibitor of HSP90 [57], and hypothesized that the activity of the 26S proteasome would decrease. However, the proteasome activity increased in the S cells and did not change in the R and T cells (Figure 9B). These results are difficult to explain unambiguously, but the following considerations seem relevant. When HSP90 is blocked with AADG, the 19S caps, which are required for the recognition of K48 polyubiquitinated proteins, do not attach to the 20S cores of the proteasome [73]. However, the 20S core exhibits proteolytic activity without specificity for ubiquitinated proteins [52]. The specific 26S proteasome substrate Suc-LLVY-AMC [58] is a mimetic that docks at the proteolytic cleavage site but does not reflect the ubiquitinated protein form; therefore, it can be cleaved directly by the 20S proteasome core. Then, in cells treated with AADG, the 20S core predominated over complete 26S proteasomes. These cores can cleave Suc-LLVY-AMC faster than the proteasome. The possible acceptance of this hypothesis is enhanced by the fact that geldanamycin increased the relative activity of the 20S proteasome core [73]. In R and T cells with a higher content of HSP90, the inhibitory effect of AADG was less pronounced; therefore, we did not observe an acceleration of Suc-LLVY-AMC cleavage.

## 4. Materials and Methods

### 4.1. Cells and Cultivation Conditions

The parental P-gp-negative mouse leukemia L1210 cell line (ACC-123 was obtained from the Leibniz-Institut DSMZ-Deutsche Sammlung von Mikroorganismen und Zellkulturen GmbH, Braunschweig, Germany) and is herein referred to as the S cell line. The P-gp-positive cell variants, R cells, were obtained by selecting S cells with vincristine. The P-gp-positive T cell variant was obtained by transfection with plasmid 10,957 obtained from Addgene (Watertown, MA, USA) containing cDNA for full-length P-gp (pHaMDRwt [74]). These three cell lines (the characterization of which is described in previous articles [13,17,18,19,20,25,61]) were used in the present study. The cells were cultured in RPMI 1640 medium containing 8% fetal bovine serum and 20 μg/l gentamicin (from Gibco, Langley, OK, USA) in a humidified atmosphere with 5% CO_2_ in air at 37 °C. These L1210 cell variants were cultivated in the presence of 5 nM BOR (from the Merck group via MERCK spol. S.r.o., Bratislava, Slovak Republic) for different time intervals (4, 8, and 24 h) prior to the experiment.

### 4.2. Cell Viability Tested by MTT Assay

Cells (5 × 10^4^ cells/well) were cultured in the presence or absence of either BOR (1–100 nM) or vincristine (1–1000 nM), which was administered directly into 200 μL of culture medium in 96-well cell culture plates. After 48 h, cell viability was assessed by MTT assay based on the reduction of MTT ([3-(4,5-dimethyldiazol-2-yl)-2,5-diphenyltetrazolium bromide) to insoluble purple formazan, the crystals of which were subsequently dissolved by dimethyl sulfoxide. Details of the assay are described elsewhere [25]. Absorbance at 540 nm was measured using a Universal Microplate mQuant spectrophotometer (BioTek Instruments, Inc., Winooski, VT, USA). 

### 4.3. Measurement of P-gp Transport Activity by Calcein/AM Retention Assay

R and T cells (5 × 10^5^) were washed twice in PBS containing 0.1% bovine serum albumin and then resuspended in 500 μL of the same buffer in the absence or presence of BOR (1 and 10 nM). Calcein/AM to a final concentration of 0.1 μM and PI to a final concentration of 0.9 μM (all from Sigma-Aldrich, St. Louis, MO, USA) were added directly to the buffer, and the samples were incubated for 20 min at 37 °C. After incubation, the cells were washed twice with ice-cold PBS. Fluorescence was measured using an Accuri C6 flow cytometer (BD Bioscience, San Jose, CA, USA). Details about the measurements are described elsewhere [17,20]. Only viable cells that were not stained with propidium iodide were evaluated. The proportion of dead cells that were stained with PI never exceeded 5%.

### 4.4. Monitoring of Cell Cycle Progression

S, R, and T cells (10^6^ cells per ml) were cultured for 4, 8, and 24 h with or without 5 nM BOR under standard culture conditions. The cells were washed with PBS and then fixed for 1 h in ice-cold 70% (*v*/*v*) ethanol. In samples of these cells, RNA was eliminated by incubation with RNase A (Thermo Fisher Scientific, Waltham, MA, USA) for 30 min at 37 °C, and then, the cellular DNA content was visualized with PI staining. The samples were analyzed by flow cytometry using an Accuri C6 flow cytometer. To exclude doublets, the doublet discrimination mode was used by evaluating the PI fluorescence signal by peak width (FL2-W) and area (FL2-A).

### 4.5. Detection of Gene Expression by qRT-PCR

Total mRNA was isolated from S, R, and T cells using TRI reagent (Molecular Research Center, Inc. Cincinnati, OH, USA) according to the manufacturer’s instructions. Two micrograms of DNAse I (Thermo Fisher Scientific, Bremen, Germany) was used to remove DNA from each sample. The total RNA in the samples thus treated was subjected to reverse transcription using the RevertAid-H Minus cDNA certified synthesis kit (Thermo Fisher Scientific, Bremen, Germany) according to the manufacturer’s protocol. Primers (the sequences and sizes of PCR products are summarized in Table 1) and cDNA samples were prepared for qPCR with iTaq Universal SYBR Green Super Mix (Bio-Rad Laboratories, Philadelphia, PA, USA). For PCR, the CFX96 Real-Time System C1000 Touch Thermal Cycler (Bio-Rad, Laboratories, USA) was used either for initial denaturation at 95 °C for 10 min or for 39 cycles at 95 °C for 15 s and at 59 °C for 30 s. The relative amount of each transcript was calculated by a standard curve generated from cycle thresholds for the cDNA samples and normalized to the amount of β-actin. Polymerase chain reaction (PCR) was performed in triplicate for each sample, and all experiments were repeated twice. Significance was established by unpaired Student’s *t*-test. The data were analyzed with Bio-Rad CFX96T software. Baseline levels for each gene were computed automatically. The results were quantified relative to the cycle threshold value according to the formula ΔΔCt = ΔCtsample−ΔCthousekeeper.

### 4.6. Western Blotting

The cell protein contents were determined by Western blotting using the following protocol: Cells were washed in PBS and then lysed by SoluLyse reagent with protease inhibitor cocktail (from Sigma-Aldrich, St. Louis, MO, USA) according to the manufacturer’s protocol. Protein lysates were applied to electrophoresis gels (30 µg per lane) and then separated by SDS–PAGE using a Mini-PROTEAN^®^ Electrophoresis System (Bio-Rad Laboratories, Philadelphia, PA, USA). The proteins were transferred by electroblotting to PVDF membranes (GE Healthcare Europe GmbH, Vienna, Austria). Protein bands were detected using specific primary antibodies against Hsp90α and HSP90αβ and GAPDH (all obtained from Santa Cruz Biotechnology, Dallas, TX, USA), Hsp90β, the 26S proteasome, polyubiquitinated proteins with K63 linkage, and polyubiquitinated proteins with K48 linkage (all obtained from Abcam Cambridge, United Kingdom). Anti-mouse/anti-rabbit antibodies conjugated with horseradish peroxidase (obtained from Santa Cruz Biotechnology, Dallas, TX, USA) were used as secondary antibodies. The protein bands were visualized with an ECL detection system (GE Healthcare Europe GmbH, Vienna, Austria) using an Amersham Imager 600 (GE Healthcare Europe GmbH, Vienna, Austria). Broad-range protein molecular weight markers (Thermo Fisher Scientific, Bremen, Germany) were used for molecular weight estimations. The optical density of the protein bands was quantified by densitometry using ImageAmersham™ image analysis software (GE Healthcare Europe GmbH, Vienna, Austria). All estimations were performed in triplicate, and the optical densities were normalized to GAPDH as an internal standard. Significance was established by unpaired Student’s *t*-test.

### 4.7. Visualization of HSP90α-and β-Immunoreactive Proteins in S, R, and T Cells by Immunofluorescence Confocal Microscopy

After culturing, the cells were washed and resuspended in PBS, and then, the cells were transferred to poly-*L*-lysine slides (Menzel Glaser, (Thermo Fisher Scientific, Bremen, Germany). Slide-bound cells were washed twice in PBS and then fixed with 1% paraformaldehyde in PBS for 10 min. After fixation, the cells were permeabilized with 0.1% Triton-X 100 in PBS, washed in PBS, and blocked with 1% BSA in PBS for 1 h at 4 °C. The cells were then incubated with specific antibodies against both HSP90α and HSP90β (described in Section 4.6) for 1 h at 4 °C in PBS containing 1% BSA. The cells were washed twice in PBS containing 1% BSA, and donkey anti-rabbit antibody linked with Alexa Fluor 488 (Life Technologies Corporation, Wilsonville, OR, USA) in PBS containing 1% BSA was applied to the cells and incubated for 1 h at 4 °C. The samples were washed twice in PBS containing 1% BSA, and the cells were then additionally labeled with 10 mg/L of 4′-6-diamidino-2-phenylindole (DAPI, Sigma-Aldrich, St. Louis, MO, USA) in PBS for nuclei visualization. Finally, coverslips were mounted on slides with mounting medium (80% glycerol), and the samples were observed using a Leica TCS SP8 AOBS confocal microscope (Leica Microsystems, Wetzlar, Germany).

### 4.8. Immunoprecipitation of the 26S Proteasome and HSP90α or HSP90β

L1210 cell variants were incubated with or without 5 nM BOR for 24 h. Subsequently, cells were harvested, and whole cell lysates were prepared by homogenization in SoluLyse as described in Section 4.6. The isolated proteins were used for immunoprecipitation according to the following protocol. Proteins (60 μg, determined by Lowry assay) were dissolved to a final volume of 300 μL using 50 mM Tris-HCl (pH 7.0) containing antibodies against either the 26S proteasome or HSP90α (described in Section 4.6). After 2 h of incubation at 4 °C, 20 μL of protein A/G PLUS-agarose (Santa Cruz Biotechnology, Dallas, TX, USA) was added, and the mixture was incubated overnight at 4 °C. The agarose was then pelleted by centrifugation (10 min at 10,000 rpm) at 4 °C and washed twice with 50 mM Tris-HCl buffer. The agarose-bound proteins were solubilized directly in SDS-PAGE sample buffer, loaded onto a 10% gel, and electrophoretically separated. The proteins were then electroblotted onto a PVDF membrane, and the presence of 26S proteasome, HSP90α, and HSP90β in the immunoprecipitate was detected using the primary and secondary antibodies described in Section 4.4. A rabbit IgG light chain secondary signal was used as an internal standard.

### 4.9. Proteasome Activity Assay

The proteasome activity assay was performed with 2 × 10^6^ cells per well. All variants of L1210 cells were cultivated either for 4 h in the absence or presence of 5 nM BOR or 1 μM AADG (17-N-allylamino-17-demethoxygeldanamycin, Sigma-Aldrich, St. Louis, MO, USA, an inhibitor of HSP90) in cultivation medium for 24 h. Proteasome activity in the samples was measured using an ab107921 kit (Abcam Cambridge, United Kingdom) according to the manufacturer’s instructions. All details of the assay were described previously [25].

### 4.10. Statistical Analysis and Data Processing

Numerical data are expressed as the mean ± SD of three independent measurements. Statistical significance was assessed using an unpaired Student’s *t*-test using SigmaPlot 8.0 software (Systat Software, Inc., San Jose, CA, USA).

Line dependences were subjected to linear regression and were characterized by a 95% confidence interval using SigmaPlot.

Dose–response curves were fitted by the function of exponential decay (Equation (1) [25]) by nonlinear regression using SigmaPlot.

(1) $$N=N_{O}\times e^{\left[\ln\left(0.5\right)\times \left(\frac{c}{{\mathrm{IC}}_{50}}\right)\right]}$$

where N represents the MTT signal in the presence of the respective substances at concentration c and N_O_ represents the MTT signal in the absence of any substance. IC_50_ is the mean lethal concentration of substances when N = 0.5 × N0.

## 5. Conclusions

**Figure 10:**
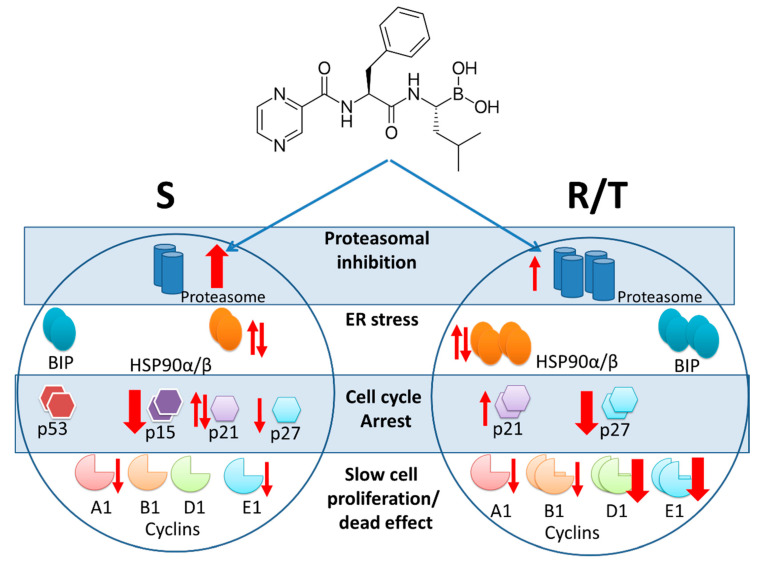
Scheme of the effect of bortezomib on S, R, and T cells. P-gp positive R and T cells have increased expression levels of the proteasome subunits, HSP90α/β, p21, and p27 and cyclins B1, D1, and E1 as P-gp negative S cells. In contrast, p15 expression is observed in S cells only. In addition, there is significantly more proapotically acting p53 in S cells, which is found in small amounts in R and T cells [61]. In contrast, R and T cells have higher levels of GRP78/BIP, which is a blocker of endoplasmic reticulum stress receptors [13]. These characteristics are responsible for the differences in proliferation between S cells on the one hand and R and T cells on the other hand. They also explain the lower apoptotic response of R and T cells to toxic stress [22] or to endoplasmic reticulum stress [13]. Bortezomib administered at a concentration of 5 nM causes a strong increase in the expression of proteasome subunits and a strong decrease in p15 expression in S cells over 24 h. The expression of cyclins A1 and E1 as well as p27 decreases less intensively in these cell variants and we did not show a clear trend with HSP90α/β and p21 protein. In contrast, the expression of cyclins D1 and E1 as well as the p27 protein decreases strongly in R and T cells during 24 h after botezomib administration. A slight decrease in expression of cyclins A1 and B1 and, conversely, a slight increase in p21 protein and proteasome subunits was observed in these cell variants. As in S cells, we did not observe a clear trend in HSP90α/β and p21 expressions in R and T cells. The changes induced by botezomib treatment in P-gp-negative S cells and P-gp-positive R and T cells appear to attenuate differences in the original observed characteristics present before treatment.

Variants of P-gp-positive mouse L1210 leukemia cells, R and T cells, are sensitive to BOR at nanomolar concentrations, similar to their P-gp-negative S cell counterparts. P-gp transport activity does not appear to limit the effect of BOR on R or T cells. However, we found various changes between the R and T cells on the one hand and S cells on the other hand that could protect P-gp-positive cells from the effect of BOR. These changes (summarized on Figure 10) include differences in cell cycle progression between P-gp-positive and P-gp-negative cells. BOR induced less pronounced G2/M phase cell cycle arrest in the R and T cells than in the S cells. These features are associated with altered expression levels of genes encoding CDKs, their activating cyclins, and/or their protein inhibitors in the R and T cells compared to S cells.

When comparing P-gp-positive R and T cells with P-gp-negative S cells, altered expression levels are observed in deubiquitinases, 26S proteasome subunits or HSP90. After incubating the cells with BOR and subsequent removal of BOR by extensive washing, the proteasome activity in the S cells is reduced more than in the R or T cells. However, these changes, although statistically significant, are not sufficient to alter the overall sensitivity of the cells to BOR.

## Figures and Tables

**Table 1:** The primer sequences.

Gene	Forward Primer	Revers Primer	bp
*Actb*	5′-TCG CCA TGG ATG ACG ATA-3′	5′-CAC GAT GGA GGG GAA TAC AG-3′	110
Human *ABCB1*	5′-GCA ATG GAG GAG CAA AGA AG-3′	5′-CCA AAG TTC CCA CCA CCA TA-3′	150
Mouse *Abcb1*	5′-TGG GAA CTC TGG CTG CTA TT-3′	5′-GGC GTA CGT GGT CAT TTC TT-3′	179
*Abcc1*	5′-ACC AGC AAC CCC GAC TTT AC-3′	5′-TGG TTT TGT TGA GGT GTG TCA-3′	151
*Abcg2*	5′-CCA CGT GTT AGT ACC AAT GTC G-3′	5′-TTT CCG GAC TAG AAA CCC ACT-3′	151
*Psmb1*	5′-CGA GAT AGC CCC AAA TGC TA-3′	5′-AAG AAG CGC CGT GAG TAC AG-3′	194
*Psmb2*	5′-GTC ACC CCC AGC TCA GTC-3′	5′-GGA AGC GAC GAG GAC ATA GT-3′	153
*Psmb5*	5′-GCC ATC TAC CAA GCC ACC TA-3′	5′-TAG CCA TGG AGA CCC ACC TA-3′	193
*Psma7*	5′-GTT GGT GTT CGA GGA AAG GA-3′	5′-GTC ACT GGG TCC TCC ACT GT-3′	212
*Usp7*	5′-AGC AAC GCA GAA GAG GAC AT-3′	5′-TTT GGT GTG GTC TGT CTG GA-3′	181
*Usp28*	5′-CCA GTC ACG ACA CAA CTG CT-3′	5′-TCC AGG AGA CTC AAG GCA AT-3′	139
*Usp48*	5′-GAT CCA TGG GGG AAA AGA TT-3′	5′-TGC TGC TGC CCA GTA TCT AA-3′	144
*Usp1*	5′-AAG GGA AGC TGC AAA GAA GA-3′	5′-GCC TTG GCT GTG TAG CAA GT-3′	145
*Usp2*	5′-GTG GTG AGC CCA TCT GAG TT-3′	5′-GTG GAG ACC ATC CAG AAG GA-3′	114
*Uchl5*	5′-TGG TCC AGG ACT CCA GAC TT-3′	5′-TGC ACA TCT TGA TGC GTA CA-3′	115
*p15*	5′-AGG ACG CTC ACC GAA GCT A-3′	5′-CTG TGG CAG AAA TGG TCC TT-3′	100
*p16*	5′-CAA CGT TCA CGT AGC AGC TC-3′	5′-ACC AGC GTG TCC AGG AAG-3′	120
*p27*	5′-AGT CAG CGC AAG TGG AAT TT-3′	5′-AGT AGA ACT CGG GCA AGC TG-3′	100
*p21 t1*	5′-TCC ACA GCG ATA TCC AGA CA-3′	5′-ATG AGC GCA TCG CAA TCA C-3′	148
*p21 t2*	5′-TTA AGG ACG TCC CAC TTT GC-3′	5′-AGA CAA CGG CAC ACT TTG CT-3′	107
*CycB1*	5′-GGT GAC TTC GCC TTT GTG AC-3′	5′-CTA CGG AGG AAG TGC AGA GG-3′	125
*CycD1*	5′-AGC AGA AGT GCG AAG AGG AG-3′	5′-CAA GGG AAT GGT CTC CTT CA-3′	149
*CycE1*	5′-GGA AAA TCA GAC CAC CCA GA-3′	5′-AGG ATG ACG CTG CAG AAA GT-3′	131
*CycA1*	5′-ACA CAG ACC CAA GGC TCA CT-3′	5′-ACA GGG TCT CTG TGC GAA GT-3′	122
*Cdk2*	5′-CAT TCC TCT TCC CCT CAT CA-3′	5′-TAA GCA GGT TCT GGG GCT TA-3′	110
*Cdk4*	5′-TAT GAA CCC GTG GCT GAA AT-3′	5′-TCC AGC TGC TCC TCC ATT AG-3′	123
*Cdk6*	5′-AGA AGG TCG GTC CGT CTA GC-3′	5′-ACT CAG GCT GTC CTT CTC CA-3′	132
*Cdk1*	5′-TTG AAA GCG AGG AAG AAG GA-3′	5′-TCC ATG GAC AGG AAC TCA AA-3′	148
*Hsp90aa*	5′-GGG AGC TCA TCT CCA ATT CA-3′	5′-ATT GAT GTG CAG CTC CTT CC-3′	101
*Hsp90ab*	5′-GAT TGT CAC CTT TTC AAC CTT CTT-3′	5′-TGA CCT CCT TGT CAG AGT ATG TGT-3′	344

## Data Availability

Additional data as well as resistant variants of L1210 cells are available from the authors.

## Supplementary Materials

The following are available online at https://www.mdpi.com/article/10.3390/ijms22115504/s1, Table S1: Characterization of S, R and T variants of L1210 cells. Figure S1: Structures of bortezomib—proteasome inhibitor, 17-(allylamino)-17-demethoxy-geldanamycin—HSP90 inhibitor, Suc-LLVY-AMC—Proteasome substrate and MG-132 proteasome inhibitor. Figure S2: Effect of bortezomib and vincristine on the viability of S, R and T variants of L1210 cells. Figure S3: Effect of bortezomib on expression of MRP1 and BCRP in S, R and T cells. Figure S4: Histograms of the fluorescence cytometry analysis used to detect cell cycle progression based on quantification of cellular DNA stained with propidium iodide. Figure S5: Expression of the Cdkn1a gene encoding the p21 protein in S, R and T cells. Figure S6: Immunodetection of HSP90 by an antibody against both HSP90α and HSP90β. Figure S7: Measurements of 26S proteasome activity with an ab107921 kit.

## Abbreviations

19S	Regulatory cap of proteasome
20S	Catalytic core of proteasome
26S	Completed proteasome
AADG	17-(allylamino)-17-demethoxygeldanamycin
ABC	ATP-binding cassette
ABCB1	B1 member of ABC transporter family, P-gp
ABCC1	C1 member of ABC transporter family, MRP1
ABCC/ABCG	C and G subfamily of ABC transporter
Akt	Serine/threonine specific protein kinase B (PKB)
ALL	Acute lymphoblastic leukemia
AMC	7-amino-4-methylcoumarin
ATP	Adenosine triphosphate
BiP	Binding immunoglobulin protein
BOR	Bortezomib
BSA	Bovine serum albumin
Calcein/AM	Acetoxymethyl-calcein
CC	Cell cycle
*Ccna1, b1,d1,e1*	Mouse gene encoding cyclin A1, B1, D1, and E1, respectively
CDK(s)	Cyclin-dependent kinase(s)
*Cdk1, 2, 4, 6*	Mouse gene encoding CDK1, 2, 4, and 6, respectively
CDK1, 2, 4, 6	Cyclin-dependent kinase 1, 2, 4, and 6, respectively
CIP	CDK-interacting protein, inhibitor of CDK
CKIs	Cyclin-dependent kinase inhibitors
DAPI	4′,6-diamidino-2-phenylindole, fluorescent stain
DSMZ	German Collection of Microorganisms and Cell Culture
ECL	Enhanced chemiluminescence
EMA	European medicines agency
ER	Endoplasmic reticulum
FAV	Fluorescein isothiocyanate labeled annexin V
FDA	U.S. Food and Drug Administration
GAPDH	Glyceraldehyde 3-phosphate dehydrogenase
GRP78	Glucose-regulated protein 78-kda (bip)
HSP90	Heat shock protein 90
*Hsp90aa, ab*	Mouse gene encoding HSP90α and β, respectively
HSP90α, β	Heat shock protein 90α and β, respectively
IC50	Half maximal inhibitory concentration
IgG	Immunoglobulin G
INK4	Inhibitor of CDK4
K48	Lysine 48
K63	Lysine 63
KIP	Kinase inhibitory protein, inhibitor of CDK
L1210	Mouse lymphocytic leukemia cell line
MDR	Multidrug resistance
MG-132	Inhibitor of proteasome
MTT	Methylthiazolyldiphenyl-tetrazolium bromide
p15, 16	CDK4/6-inhibitors known also as INK4
p21, 27, 57	CDK-interacting protein/kinase inhibitory protein (CIP/KIP)
PAGE	Polyacrylamide gel electrophoresis
PBS	Phosphate buffered saline
PCR	Polymerase chain reaction
PFD	Paraformaldehyde
P-gp	P-glycoprotein
PI	Propidium iodide
PNCA	Proliferating cell nuclear antigen
*Psma7*	Mouse gene encoding proteasome subunit α7
*Psmb1, b2, b5*	Mouse gene encoding proteasome β1, β2, and β5, respectively
PVDF	Polyvinylidene fluoride, special plastic
qRT-PCR	Quantitative reverse transcription-polymerase chain reaction
R	P-gp positive L1210 cells obtained by selectin with VCR
RPMI 1640	Roswell Park Memorial Institute 1640 medium
S	Parental L1210cells
SDS	Sodium dodecyl sulfate
T	P-gp positive L1210 cells obtained by transfection with P-gp
TRAF2	Tumor necrosis factor receptor-associated factor 2
Ubi	Ubiquitin
UPR	Unfolded protein response
UCHL5	Ubiquitin C-Terminal Hydrolase L5
USP1	Ubiquitin-specific protease 1
USP2	Ubiquitin-specific protease 2
USP28	Ubiquitin-specific protease 28
USP48	Ubiquitin-specific protease 48
USP7	Ubiquitin-specific protease 7
VCR	Vincristine
YB-1	Y box binding protein 1
