# Supplementary material for: Insight into Bortezomib Focusing on Its Efficacy against P-gp-Positive MDR Leukemia Cells
Source: Int J Mol Sci. 2021 May 23;22(11):5504. doi: 10.3390/ijms22115504 (PMC8197160; doi:10.3390/ijms22115504)
Supplement: Supplementary file 1 [file ijms-22-05504-s001.zip › ijms-1210800-supplementary.pdf]

International Journal of *Molecular Sciences*

MDPI

Supplementary files

Kyca et al., "Insight into bortezomib focusing on its efficacy against P-gp-positive MDR leukemia cells."

Characterization of S, R and T variants of L1210 cells

Table S1. Characteristicd of S, R and T vatiants of L1210 cells.

Cell variants	R	T	Reference
Fold difference from S in:			
Cell resistance			
Resitance to			
cisPlatin	≈2	≈2	[1,2]
Doxorubicin	<150 times[2]	<150 times[2]	[2]
Vincristine	<150 times[2]	<150 times[2]	[2]
Tunicamycin	Maximally 3 passages[1]	Unlimited number of passages[1]	[3-5]
ABC transporters			
P-gp mRNA	<150 times[2]	<150 times[2]	[6,7]
P-gp protein	<500< times[2]	<500 < times[2]	[6,7]
Pgp activity measured with calcein retention	positive	Positive	[4,6,7]
BCRP mRNA	≈4 times	≈1.2 times	
BCRP protein	≈1.5 times	≈0.8 times	[6]
MRP1 mRNA	<3 times	<5 times	
Endoplasmic reticulum stress related proteins			
GRP78/BiP mRNA	≈3 times[2]	≈2.4 times[2]	
GRP78/BiP protein	≈4 times[2]	≈7 times[2]	
PERK mRNA	≈1.2 times	≈1.4 times	
PERK protein	≈2.2 times	≈2.6 times	
IRE1α mRNA	≈1.6 times	≈1.2 times	[5,6]
IRE1α protein	≈1.6 times	≈1.5 times	
ATF6 mRNA	≈1.4 times	≈1.5 times	
ATF6 90 kDa protein	≈1.1 times	≈1.6 times	
ATF6 50 kDa protein	≈4.1 times[2]	≈3.8 times[2]	

The red data showed a statistically significant difference from S cells at least at the level of p; [1] In medium containing 100 nM tunicamycin; [2] The difference from S cells is responsible for the change in the cellular response to stress stimuli.

Conclusion: R and T are cell variants with multiple resistance associated with P-gp overexpression. These cells are resistant not only to P-gp-related drugs, but additional resistance to other substances such as cysplatin and tunicamicin occured. The massive overexpression of the P-gp together with changes in various other molecular properties is responsible for the resistant character of the cells. GRP78/BiP overexpression is responsible for the altered response of P-gp positive cells to ER stress compared to S cells [5,6]. This protein binds to unfolded proteins within ER and is therefore released from the blocking bonds with ER stress receptors. Elevated levels of 50 kDa ATF6 was

detected in R and T cells. This proteins results from cleavage from the 90 kDa ATF6 receptor and is transcription factor active in cell response to ER stress.

Structures of bortezomib – proteasome inhibitor, 17-(allylamino)-17-demethoxy-geldanamycin – HSP90 inhibitor, Suc-LLVY-AMC – Proteasome substrate and MG-132 proteasome inhibitor.

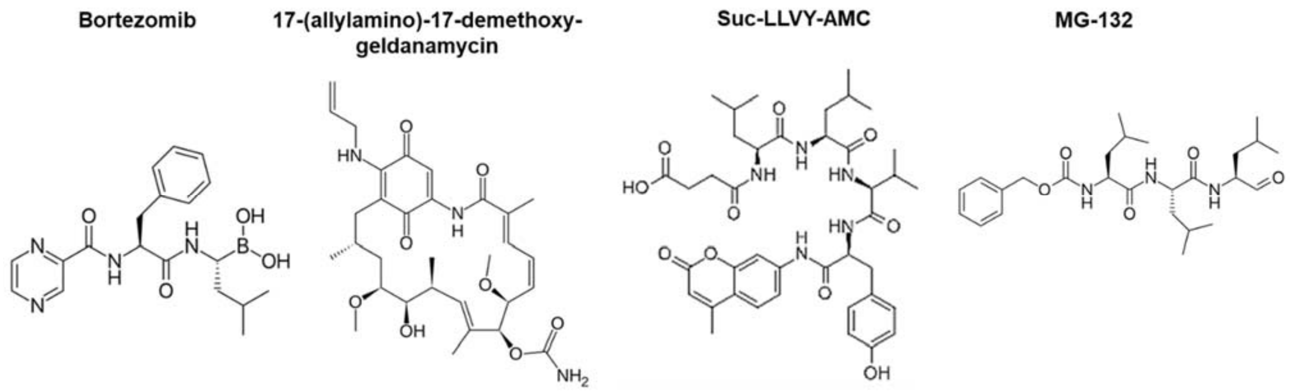

Figure S1: Bortezomib — systematic name: N-[(1R)-1-(dihydroxyboryl)-3-methylbutyl]-Nα-(2-pyrazinylcarbonyl)-L-phenylalaninamide. **17-(allylamino)-17-demethoxygeldanamycin** — systematic name: (4Z,6Z,8S,9S,10Z,12S,13R,14S,16R)-19-(Allylamino)-13-hydroxy-8,14-dimethoxy-4,10,12,16-tetramethyl-3,20,22-trioxo-2-azabicyclo[16.3.1]docosa-1(21),4,6,10,18-pentaen-9-yl carbamate. **Suc-LLVY-AMC** — systematic name: N-(3-carboxypropanoyl)-L-leucyl-L-leucyl-L-valyl-N-(4-methyl-2-oxo-2H-chromen-7-yl)-L-tyrosinamide. **MG-132 —** systematic name: N-[(benzyloxy)carbonyl]-L-leucyl-N-[(2S)-4-methyl-1-oxo-2-pentanyl]-L-leucinamide

Effect of bortezomib and vincristine on the viability of S, R and T variants of L1210 cells

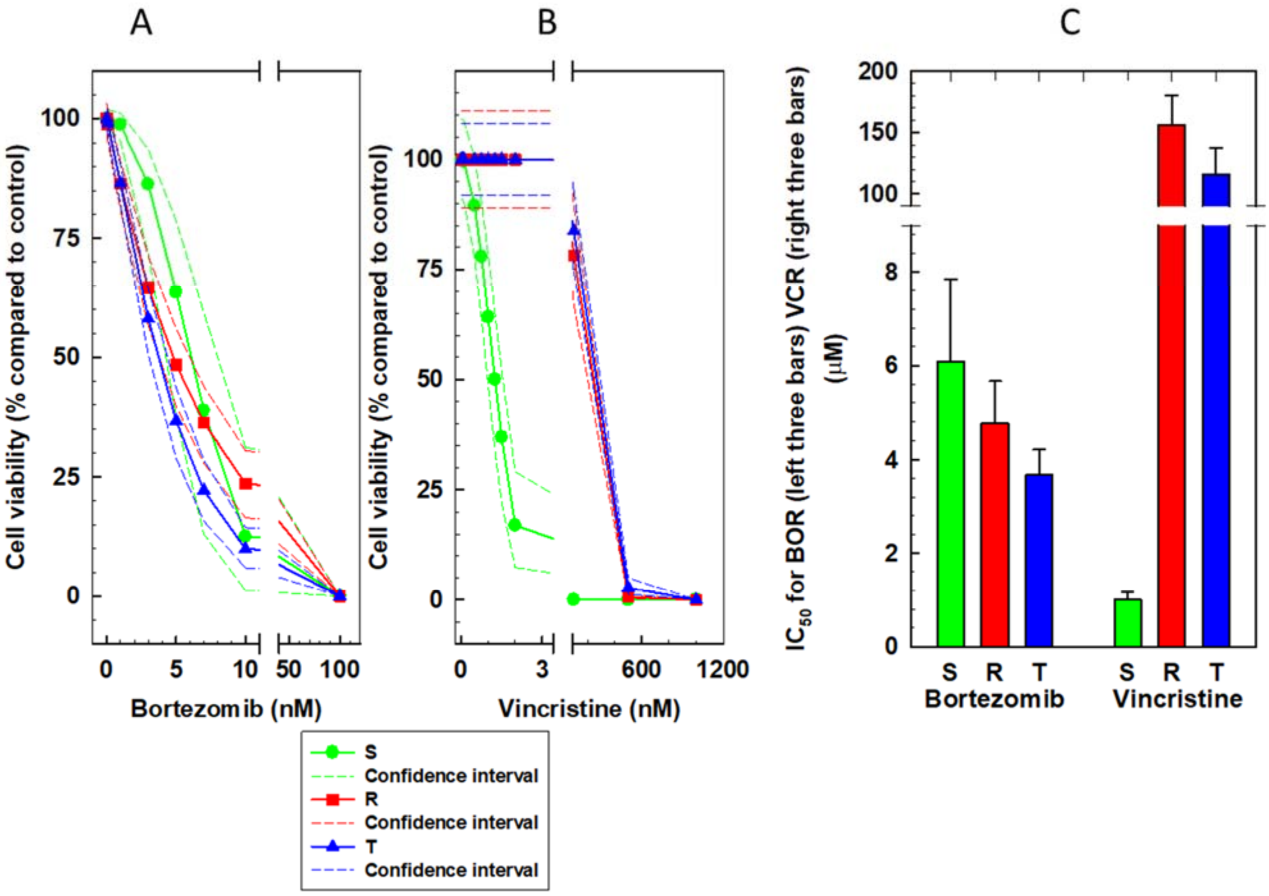

Figure S2. Effects of ConA on the viability of S, R and T cells. The number of cells without drug treatment was arbitrarily set to 100%. The data represent the mean±standard error of six independent experiments. Curves were obtained by nonlinear regression according to Eq. (1) described in section 4.2. Confidence intervals are at the 95% level. Panel A — effects of bortezomib. Panel B — effects of vincristine. Panel C — IC_{50} values of bortezomib and vincristine in proliferating S, R and T cells; the data represent the calculated value ± standard error for 40 degrees of freedom.

Conclusion: Bortezomib induces similar cell death effects in the P-gp-positive R and T variants as it induces in the P-gp-negative S variant of L1210 cells. In contrast, the R and T cells are significantly less sensitive to the typical substrate P-gp, vincristine.

Effect of bortezomib on expression of MRP1 and BCRP in S, R and T cells

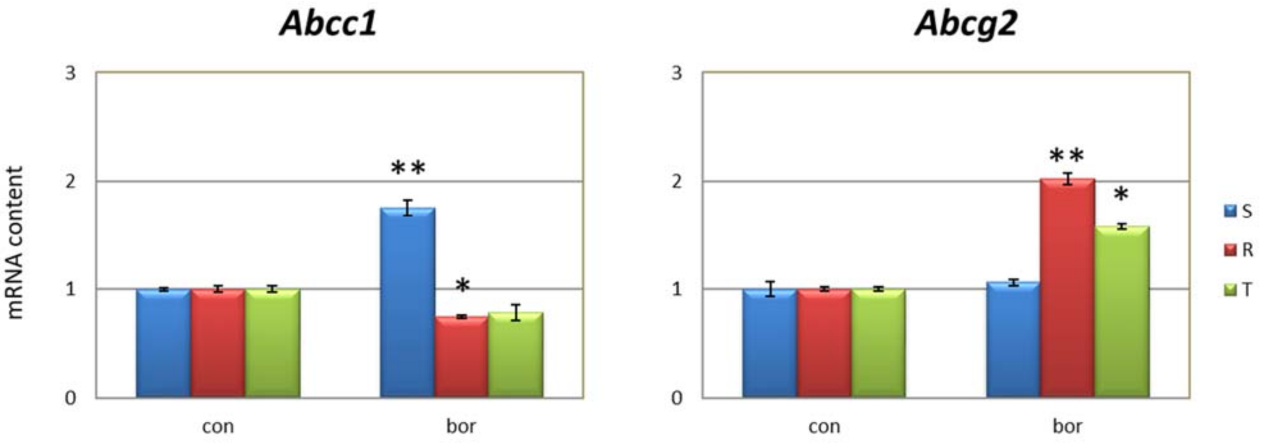

Figure S3. Effect of BOR (5 nM) on the expression of MRP1 (product of Abcc1 gene) and BCRP (product of Abcg2 gene) in S, R and T cells. β-actin was used as an internal standard and relative quantity of mRNA for cells in the absence of BOR (con) was arbitrarily taken as one. The results represent the means±SD of three independent measurements. Statistical significance: * and ** - data after BOR treatment differ from untreated control at $p < 0.02$ and $p < 0.01$, respectively.

Histograms of the fluorescence cytometry analysis used to detect cell cycle progression based on quantification of cellular DNA stained with propidium iodide.

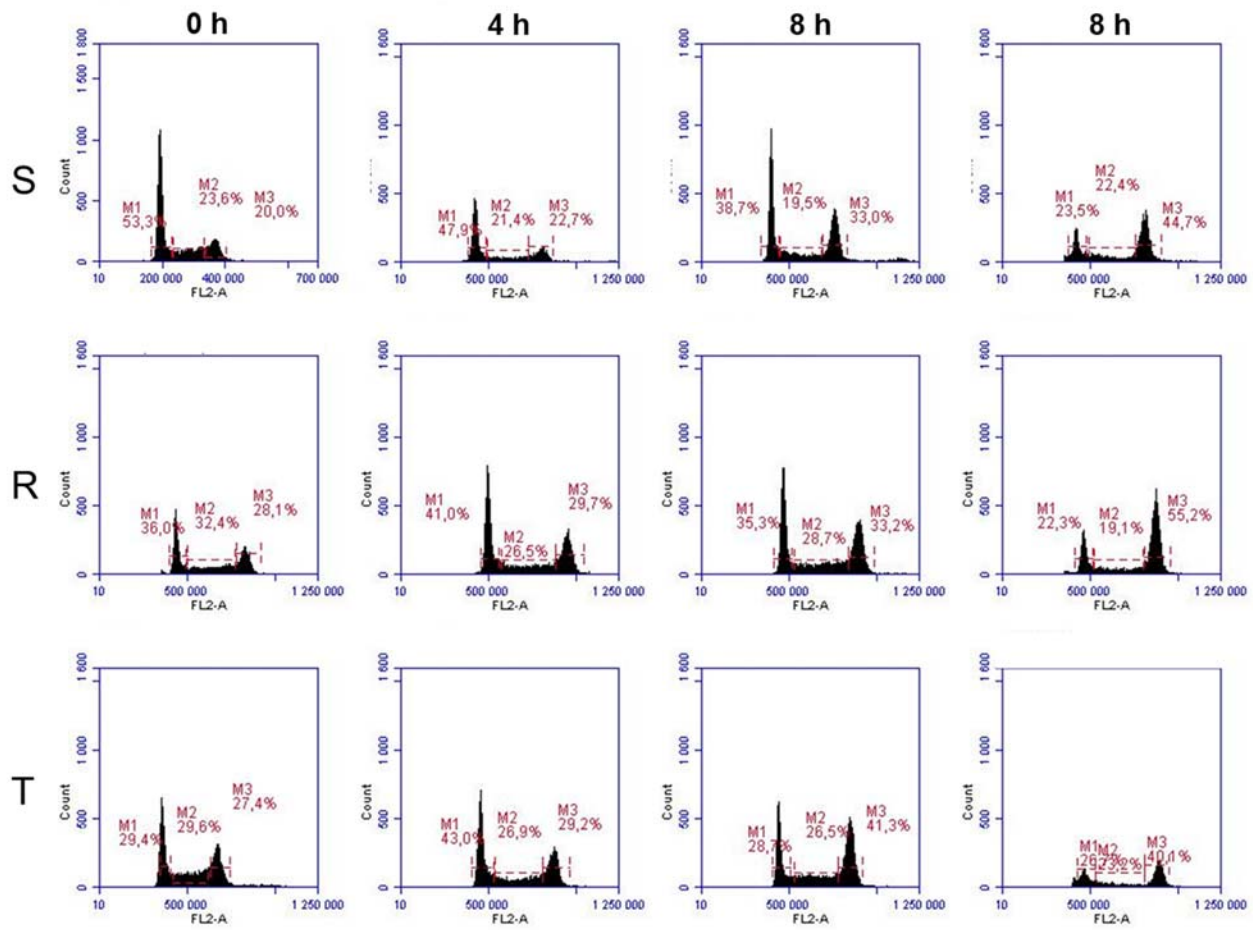

Figure S4. Fluorescence cytometry histograms of the cell cycle as detected by PI staining. S, R and T cells after incubation for a given time in medium in the presence or absence bortezomib (at concentrations of 5 nM) were assessed. The histograms are representative of three independent experiments. The M1, M2, and M3 intervals delimit subpopulations of cells in the G0/G1 phase, S phase, and G2/M phase of the cell cycle, respectively.

The average proportion of cells in the individual phases of the cell cycle was obtained with three independent measurements and used to create Figure 2 in section 2.2.

Expression of the Cdkn1a gene encoding the p21 protein in S, R and T cells

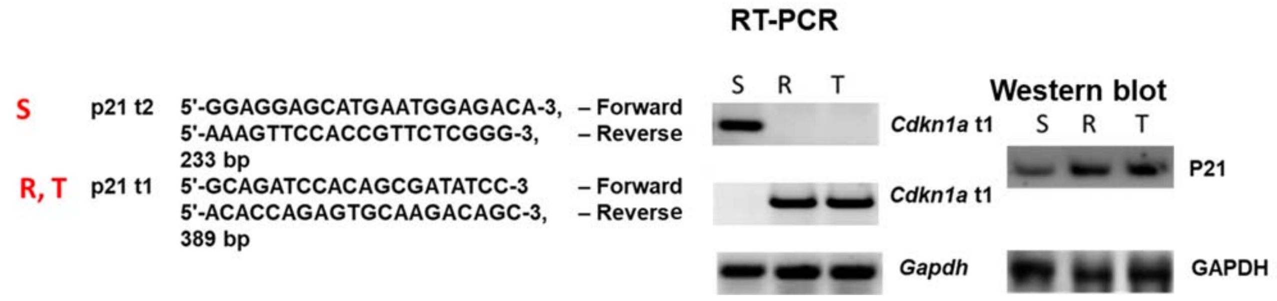

Figure S5. Expression of the *Cdkn1a* gene encoding the p21 protein in S, R and T cells. For RT-PCR detection of p21 transcripts, we searched for the gene in the GeneCards database (Weizmann Institute of Science, Rehovot, Israel), used the Ensembl genome browser [8] to determine the sequence of transcriptional variants, and designed the primers using primer3 (Whitehead Institute for Biomedical Research, Cambridge, MA, USA). Western blots were processed as described in section 4.6. The RT-PCR and Western blot data are representative of three independent measurements.

RT-PCR protocol: The cDNA samples obtained as described in section 4.5 were subjected to PCR with the primers shown in the figure according to the following protocol: After heating at 94 °C for 3 minutes to inactivate reverse transcriptase, the samples were subjected to 30 cycles of PCR with 45 sec of denaturation (at 95 °C); 30 sec of annealing (at 58 °C for p21 t2, 57 °C for p21 t1, and 56.6 °C for GAPDH) followed by a final extension at 72 °C for 10 minutes. The PCR products were separated on a 1.7% agarose gel (Invitrogen, Life Technology, Bratislava, Slovakia) and visualized with GelRed™ nucleic acid gel stain (Thermo Fisher Scientific, Bremen, Germany) using an Amersham Imager 600 (GE Healthcare Europe GmbH, Vienna, Austria).

Conclusion: Two variants of the p21 transcripts are evident in S, R and T cells: a common t2 transcript, which is present exclusively in the S cells, and an alternative t1 transcript, which is present exclusively in the R and T cells. Both transcripts encode the full length p21 protein. Considering these results, we designed Primer3 primers for qRT-PCR based on the respective transcript variants, which are given in Table 1.

Immunodetection of HSP90 by an antibody against both HSP90α and HSP90β

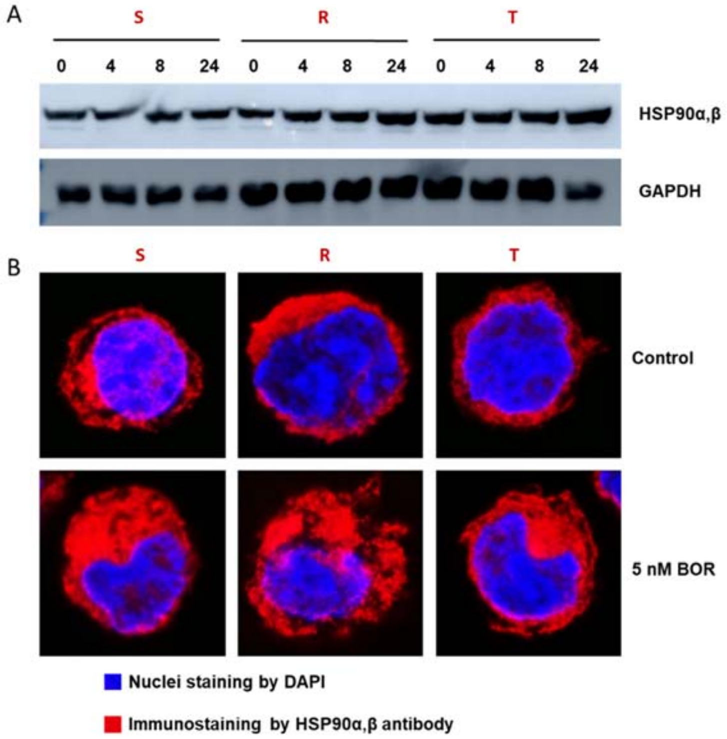

Figure S6. Detection of HSP90 by antibody against both HSP90α and HSP90β. Panel A: Levels of HSP90αβ in S, R and T cell variants as determined by Western blot analysis. The cells were incubated for 4, 8 and 24 hours in growth medium in the presence of BOR (5 nM) in a CO_2 incubator. GAPDH was used as an internal control. The data are representative of three independent measurements. Panel B: Immunofluorescence visualization of HSP90α and HSP90β in the S, R and T cells. The cells were cultivated for 24 hours in the absence or presence of BOR (5 nM) and processed according to the procedure described in section 4.7. The data are representative of six independent measurements.

Conclusions: Incubation of S, R and T cells in BOR-containing medium increased the levels of proteins interacting with the antibody against both HSP90α and HSP90β**.**

Measurements of 26S proteasome activity with an ab107921 kit.

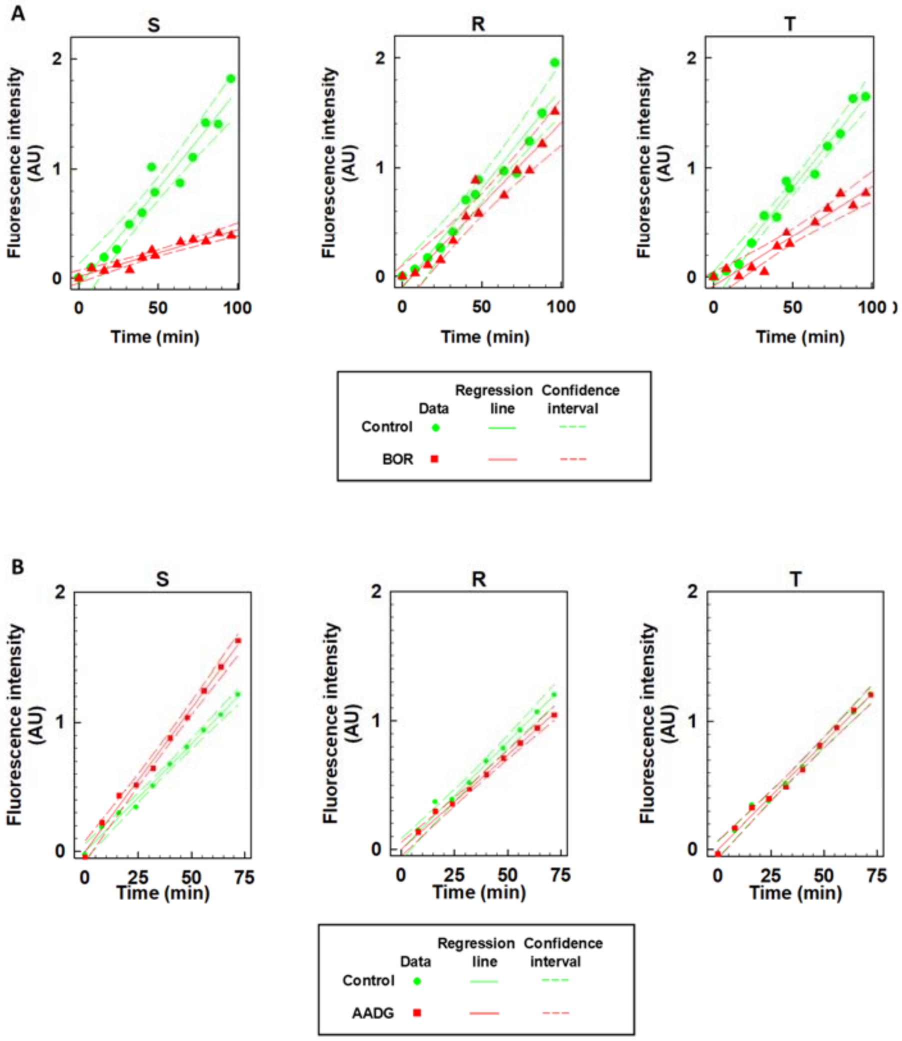

Figure S7. Time course of Suc-LLVY-AMC cleavage by the proteasome. The time courses can be described by zero-order kinetics, which is typical for enzyme reactions, for each cell variant; therefore, straight lines were plotted. The confidence intervals were at the 95% level and valid for 11 degrees of freedom. The initial velocities of Suc-LLVY-AMC cleavage by the proteasome were obtained as the slope of the respective time course and are summarized in Figure 9. Panel A: Effect of S, R and T cell incubation in medium containing BOR (5 nM) for 24 hours. Panel B: Effect of S, R and T cell incubation in medium containing AADG (1 μM) for 24 hours.

Conclusions: The time course of Suc-LLVY-AMC cleavage in homogenates of S, R and T cells during the time interval was linearly consistent with that of our previous paper [6]. This linear relationship allowed us to determine the initial velocity of the reaction as the slope of the straight line.

References

1. Gibalova, L.; Seres, M.; Rusnak, A.; Ditte, P.; Labudova, M.; Uhrik, B.; Pastorek, J.; Sedlak, J.; Breier, A.; Sulova, Z. P-glycoprotein depresses cisplatin sensitivity in l1210 cells by inhibiting cisplatin-induced caspase-3 activation. Toxicol In Vitro 2012, 26, 435-444.
2. Pavlikova, L.; Seres, M.; Imrichova, D.; Hano, M.; Rusnak, A.; Zamorova, M.; Katrlik, J.; Breier, A.; Sulova, Z. The expression of p-gp in leukemia cells is associated with cross-resistance to protein n-glycosylation inhibitor tunicamycin. Gen Physiol Biophys 2016, 35, 497-510.

3. Pavlikova, L.; Seres, M.; Hano, M.; Bohacova, V.; Sevcikova, I.; Kyca, T.; Breier, A.; Sulova, Z. L1210 cells overexpressing abcb1 drug transporters are resistant to inhibitors of the n- and o-glycosylation of proteins. Molecules 2017, 22.
4. Seres, M.; Cholujova, D.; Bubencikova, T.; Breier, A.; Sulova, Z. Tunicamycin depresses p-glycoprotein glycosylation without an effect on its membrane localization and drug efflux activity in l1210 cells. Int J Mol Sci 2011, 12, 7772-7784.
5. Seres, M.; Pavlikova, L.; Bohacova, V.; Kyca, T.; Borovska, I.; Lakatos, B.; Breier, A.; Sulova, Z. Overexpression of grp78/bip in p-glycoprotein-positive l1210 cells is responsible for altered response of cells to tunicamycin as a stressor of the endoplasmic reticulum. Cells 2020, 9.
6. Cagala, M.; Pavlikova, L.; Seres, M.; Kadlecikova, K.; Breier, A.; Sulova, Z. Development of resistance to endoplasmic reticulum stress-inducing agents in mouse leukemic l1210 cells. Molecules 2020, 25.
7. Sulova, Z.; Ditte, P.; Kurucova, T.; Polakova, E.; Rogozanova, K.; Gibalova, L.; Seres, M.; Skvarkova, L.; Sedlak, J.; Pastorek, J., et al. The presence of p-glycoprotein in l1210 cells directly induces down-regulation of cell surface saccharide targets of concanavalin a. Anticancer Res 2010, 30, 3661-3668.
8. Yates, A.D.; Achuthan, P.; Akanni, W.; Allen, J.; Allen, J.; Alvarez-Jarreta, J.; Amode, M.R.; Armean, I.M.; Azov, A.G.; Bennett, R., et al. Ensembl 2020. Nucleic Acids Res 2020, 48, D682-D688.

1. Yates, A.D.; Achuthan, P.; Akanni, W.; Allen, J.; Allen, J.; Alvarez-Jarreta, J.; Amode, M.R.; Armean, I.M.; Azov, A.G.; Bennett, R., et al. Ensembl 2020. *Nucleic Acids Res* **2020**, *48*, D682-D688, doi:10.1093/nar/gkz966.
2. Cagala, M.; Pavlikova, L.; Seres, M.; Kadlecikova, K.; Breier, A.; Sulova, Z. Development of Resistance to Endoplasmic Reticulum Stress-Inducing Agents in Mouse Leukemic L1210 Cells. *Molecules* **2020**, *25*, doi:10.3390/molecules2511217.
